# A Persistent Homology Approach to Heart Rate Variability Analysis With an Application to Sleep-Wake Classification

**DOI:** 10.3389/fphys.2021.637684

**Published:** 2021-03-01

**Authors:** Yu-Min Chung, Chuan-Shen Hu, Yu-Lun Lo, Hau-Tieng Wu

**Affiliations:** ^1^Department of Mathematics and Statistics, University of North Carolina at Greensboro, Greensboro, NC, United States; ^2^Department of Mathematics, National Taiwan Normal University, Taipei, Taiwan; ^3^Department of Thoracic Medicine, Chang Gung Memorial Hospital, Chang Gung University, School of Medicine, Taipei, Taiwan; ^4^Department of Mathematics and Department of Statistical Science, Duke University, Durham, NC, United States; ^5^Mathematics Division, National Center for Theoretical Sciences, Taipei, Taiwan

**Keywords:** persistent homology, persistence diagram, persistence statistics, sleep stage, heart rate variability

## Abstract

Persistent homology is a recently developed theory in the field of algebraic topology to study shapes of datasets. It is an effective data analysis tool that is robust to noise and has been widely applied. We demonstrate a general pipeline to apply persistent homology to study time series, particularly the instantaneous heart rate time series for the heart rate variability (HRV) analysis. The first step is capturing the shapes of time series from two different aspects—the persistent homologies and hence persistence diagrams of its sub-level set and Taken's lag map. Second, we propose a systematic and computationally efficient approach to summarize persistence diagrams, which we coined *persistence statistics*. To demonstrate our proposed method, we apply these tools to the HRV analysis and the sleep-wake, REM-NREM (rapid eyeball movement and non rapid eyeball movement) and sleep-REM-NREM classification problems. The proposed algorithm is evaluated on three different datasets via the cross-database validation scheme. The performance of our approach is better than the state-of-the-art algorithms, and the result is consistent throughout different datasets.

## 1. Introduction

Heart rate variability (HRV) is the physiological phenomenon of variation in the lengths of consecutive cardiac cycles, or the rhythm of heart rate (Draghici and Taylor, 2016). Interest in HRV has a long history (Billman, 2011), and there have been several theories describing how the heart rate rhythm, including, for example, the polyvagal theory (Porges, 2009) and the model of neurovisceral integration (Thayer and Sternberg, 2006). In short, HRV results from an integration of complicated interactions between various physiological systems and external stimuli (Vanderlei et al., 2009; Shaffer et al., 2014; Draghici and Taylor, 2016) on various scales, and the autonomic nervous system (ANS) plays a critical role (Thayer and Sternberg, 2006; Porges, 2009). A correct quantification of HRV yields dynamical information of various physiological systems and has various clinical applications (Stys and Stys, 1998), including improving diagnostic accuracy and treatment quality (Vanderlei et al., 2009).

**Figure 1:**
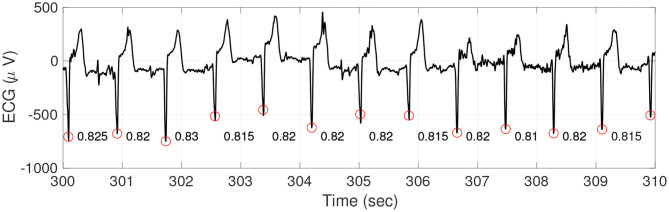
An illustration of ECG (black curve), R peaks (red circles), and RRI (the numbers between two consecutive R peaks). It is clear that the RRI changes from time to time, which form a new time series.

In practice, the heart rhythm is quantified by the time series called *instantaneous heart rate* (IHR) coming from intervals between consecutive pairs of heart beats, which is usually determined from the R peak to R peak interval (RRI) by reading the electrocardiogram (ECG). See Figure 1 for an illustration of the ECG, R peaks, and RRI. To quantify HRV, a common approach is studying various statistics of IHR. There have been a lot of efforts trying to quantify HRV, and proposed statistics could be briefly classified into four major categories—time domain approach, frequency domain approach (Task Force of the European Society of Cardiology and others, 1996), nonlinear geometric approach (Marwan et al., 2002; Voss et al., 2008), and information theory based approach (Costa et al., 2002). It is worth mentioning that while there have been a lot of researches in this direction with several proposed statistics, there is limited consensus and it is still an active research field due to the non-stationarity nature of the IHR time series (Pincus and Goldberger, 1994; Glass, 2009).

Topological data analysis (TDA) is a data analysis framework based on tools from algebraic topology (Carlsson, 2009; Epstein et al., 2011). In the past decades, its theoretical foundation has been actively established, and various algorithms have been proposed to study datasets from different fields. The basic idea underlying TDA is that the data organization can be well-captured by *counting holes*. Theoretically, the number of holes of different dimensions characterizes how the data is organized. Thus, researchers design useful statistics based on the information of holes. This simple yet powerful idea has been applied to different fields. Specifically, there have been several efforts applying TDA to analyze time series. For example, the Vietoris-Rips (VR) complex filtration and the bottleneck or Wasserstein distances among persistence diagrams are applied to study voices and body motions (Seversky et al., 2016; Venkataraman et al., 2016). A transformation of the persistence diagram, called persistence landscapes (Bubenik, 2015), has been applied to study trading records (Gidea and Katz, 2018), electroencephalogram (EEG) signals (Piangerelli et al., 2018; Wang et al., 2018; Wang et al., 2019), and cryptocurrency trend forecasting (Kim et al., 2018). Sliding Windows and 1-Persistence Scoring (Perea, 2019) offers both theoretical and practical TDA method to detect the periodicity of a time series. A brief overview of common techniques on the usages of TDA to time series is summarized in a preprint (Ravishanker and Chen, 2019). Recently, the proposed TDA tool for HRV analysis has been applied to differentiating patients with the history of transient ischemic attack and hypertension (Graff et al., 2020). However, existing TDA approaches usually suffer from computational issues, which limits its application to large scale database. Finding a computationally efficient TDA algorithm is thus critical.

In this article, motivated by the complicated interaction among different physiological systems over various scales and inter-individual variability, the need for a useful tool for the HRV analysis, and the numerical limitation of the recently developed TDA tools, we hypothesize that topological information could be useful to quantify the HRV, and propose a computationally efficient approach to analyze time series via TDA.

### 1.1. Our Contribution

Based on the flexibility of TDA tools, and due to the non-stationarity of complicated time series we commonly encounter in real life, like the IHR, we propose a systematic, principled, and computationally efficient approach to study complicated time series by the TDA tools.

**Figure 2:**
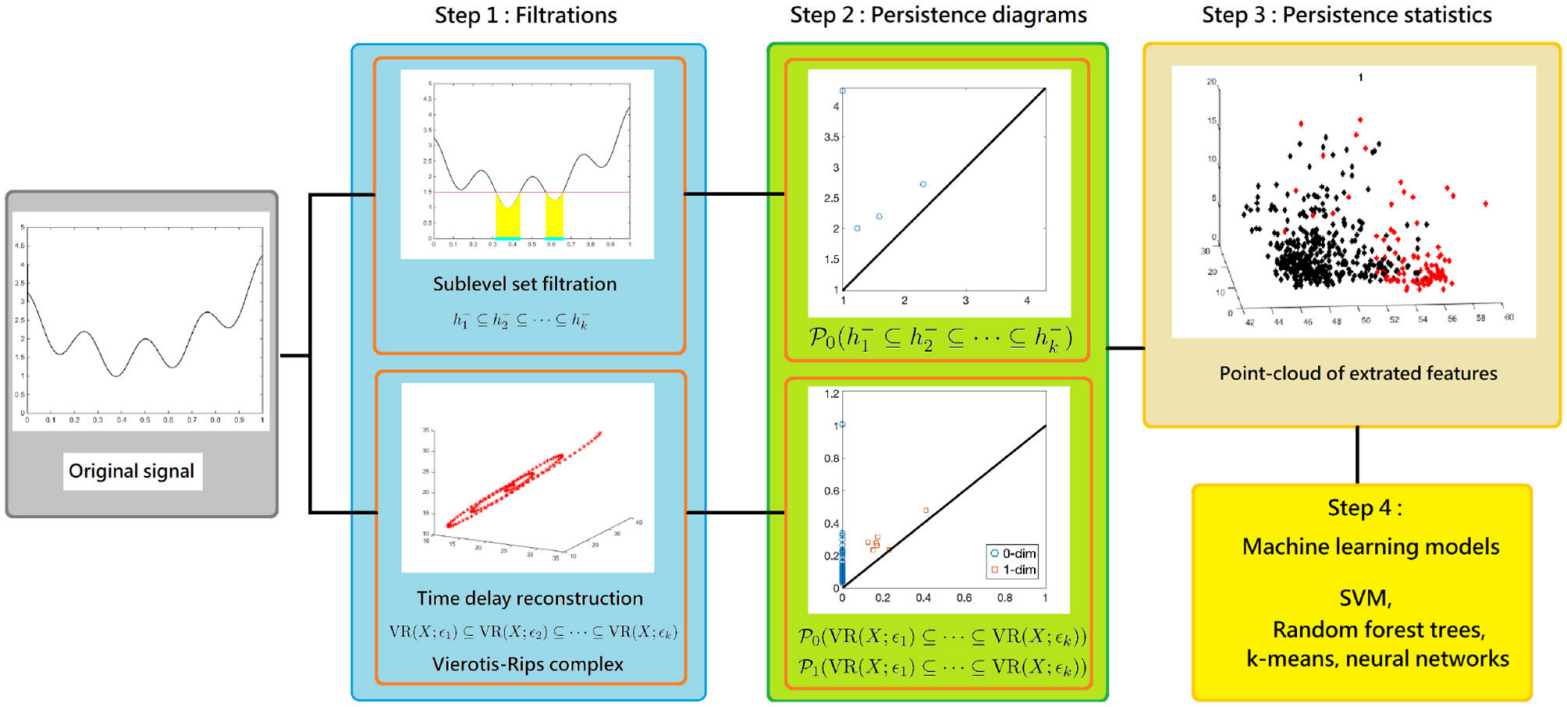
The scheme of our proposed time series analysis can be separated by three steps: constructing filtrations, computing persistence diagrams, and extracting persistence statistics as features. The features are applied to train a machine learning model for the classification purpose.

Our main scheme for studying a complicated time series is shown in Figure 2, which is divided into three steps that we will detail later. First, consider two *filtrations*, the Vietoris-Rips (VR) complex filtration of the Takens' lag map (Takens, 1981) and the sub-level set filtration of the time series, and *persistent homology*. Second, compute corresponding persistence diagrams. Finally, calculate *persistence statistics* (PS) as a novel statistic of the time series of interest. We mention that compared with existing TDA approach for time series analysis, our proposed persistence statistics features based on both sub-level set and Vietoris-Rips complexes filtrations are intuitive, straightforward to implement, and also computationally efficient.

### 1.2. Application—Sleep Dynamics

To demonstrate the usefulness of the proposed persistence statistics, we apply it to study IHR time series recorded during sleep, and use obtained statistics to classify sleep stages. Sleep is a universal recurrent physiological phenomenon. Sleep impacts the whole body, so we can read sleep via reading different physiological signals. Taking ECG into account is specifically attractive, since the ECG sensor is easy to install, and it is now widely available in mobile health devices. HRV of a subject is usually quantified by analyzing ECG, and it has been shown to be related to sleep dynamics (Zemaityte and Varoneckas, 1984; Vaughn et al., 1995; Toscani et al., 1996; Bonnet and Arand, 1997; Elsenbruch et al., 1999; Chouchou and Desseilles, 2014; Penzel et al., 2016). In other words, the heart rate rhythm provides a non-invasive window for researchers to study sleep. While there have been several studies trying to classify sleep stages based on HRV (Lewicke et al., 2008; Mendez and Matteucci, 2010; Long et al., 2012; Xiao et al., 2013; Aktaruzzaman et al., 2015; Ye et al., 2016; Malik et al., 2018), it still remains a challenging problem in the field. The challenge and difficulty of this mission can be appreciated from the reported results. In this article, we apply the proposed persistence statistics to quantify HRV during sleep, and propose a new prediction algorithm for the sleep stage; for example, an automatic classification of wake and sleep, REM and NREM, and wake, REM and NREM. We remark that while we focus on the HRV and sleep stage classification, the result indicates the potential of applying TDA-based approaches to study other complicated time series.

### 1.3. Organization

In section 2, we review the mathematical background of the persistent homology and persistence diagram. In section 3, we demonstrate two ways to use the persistent homology to study time series, and propose a new approach to summarize the persistence diagram, called the persistence statistics. The classification model based on the persistence statistics for the sleep stage classification will be discussed in detail in section 4. The discussion of our classification performance and a comparison with the state-of-arts results will be included in section 5. More technical details and numerical results are postponed to the Supplementary Material.

## 2. Mathematical Background

In this section, we describe the mathematical background, including simplicial complex, homology, filtration of sets, and the persistent homology. Although these topics can be studied in an abstract and general way (see e.g., Munkres, 1993), to enhance the readability, we present them in a relatively concrete way without losing critical information.

### 2.1. Simplicial Complexes

To investigate the complicated structure of an object, an intuitive way is to use simple objects as building blocks to approximate the original object. In TDA, the main building block is the *simplicial complex*, which we briefly recall now. See Supplementary Material (section 1.1) for more detailed mathematical background and illustrative examples.

We start with the *simplex*. Intuitively, a simplex is a “triangle” of different dimension. Let *x*_0_, *x*_1_, …, *x*_*q*_ be affinely independent points in ℝ^*d*^, where *d, q* ∈ ℕ and *d* ≥ *q*. The *q*-*simplex*, denoted by σ := 〈*x*_0_, *x*_1_, …, *x*_*q*_〉, is defined to be the convex hull of *x*_0_, *x*_1_, …, *x*_*q*_. Denote Vert(σ) := {*x*_0_, *x*_1_, …, *x*_*q*_}. Any *q*-simplex is a *q*-dimensional object consisting of lower degree simplexes. We are interested in the relation among simplexes of different dimensions. Since any *V* ⊂ Vert(σ) is also affinely independent, the convex hull of *V*, called a *face* of σ, forms a simplex of dimension |*V*| ≤ *q*, where |*V*| is the cardinality of *V*. If |*V*| = *k*, the face τ = 〈*V*〉 is called a *k*-face of σ. A *simplicial complex* K in ℝ^*d*^ is a collection of finite simplexes σ in ℝ^*d*^ so that any intersection of two arbitrary simplexes is a face to each of them; that is,

- If σ∈K and τ is a face of σ, then τ∈K;
- If $\sigma_{1},\sigma_{2}\in K$, then σ_1_ ∩ σ_2_ is a face of σ_1_ and σ_2_. In particular, $\sigma_{1}\cap \sigma_{2}\in K$.

### 2.2. Homology and Betti Numbers

In order to study the topological information of a given simplicial complex, we study relations among simplexes of different dimensions, and hence the “holes.” *Homology* and *Betti numbers* are classic subjects in the algebraic topology (Munkres, 1993), which capture “holes” of geometric objects of different dimensions. While we can discuss these topics in a more general setup, in this work, we mainly consider simplicial complexes as our target object. For example, the shape of the notation “∞” contains two 1-dimensional holes (Supplementary Figure 2C) and the empty void surrounded by the unit sphere *S*^2^ = {(*x, y, z*):*x*^2^ + *y*^2^ + *z*^2^ = 1} in ℝ^2^ is a 2-dimensional hole (Supplementary Figure 2E). Moreover, the 0-dimensional holes of an object are defined to be its disjoint connected components (Supplementary Figure 2E). See Supplementary Material (section 1.2) for more information and illustrative examples.

We need an algebraic structure of simplexes. Given *q*-simplexes σ_1_, σ_2_, …, σ_*n*_ in a simplicial complex K, define the sum over ℤ_2_ as $c=\overset{n}{\underset{i=1}{\sum }}\nu_{i}\sigma_{i}$, where ν_*i*_ ∈ ℤ_2_. This formal sum is commonly known as a *q*-*chain*. One could also define an addition operator as $\overset{n}{\underset{i=1}{\sum }}\nu_{i}\sigma_{i}+\overset{n}{\underset{i=1}{\sum }}\mu_{i}\sigma_{i}: =\overset{n}{\underset{i=1}{\sum }}\left(\nu_{i}+\mu_{i}\right)\sigma_{i}$. We consider the collection of all *q*-chains, denoted as

$$\begin{array}{l} C_{q}\left(K\right):=\{\overset{n}{\underset{i=1}{\sum }}\nu_{i}\sigma_{i}\;|\;\nu_{i}\in {\mathbb{Z}}_{2},\;\sigma_{i}\in K,\;\dim\left(\sigma_{i}\right)=q\}. \end{array}$$

One could prove that $C_{q}\left(K\right)$ is actually a vector space over ℤ_2_ with the above addition. There is a natural relation between $C_{q}\left(K\right)$ and $C_{q-1}\left(K\right)$, called the *boundary map* (Munkres, 1993, section 1.5, p. 30). The *q*th *boundary map* $\partial_{q}:C_{q}\left(K\right)\to C_{q-1}\left(K\right)$ over ℤ_2_ is defined by

$$\begin{array}{l} \partial_{q}\left(\langle x_{0},x_{1},\cdots \;,x_{q}\rangle \right):=\overset{q}{\underset{i=0}{\sum }}\langle x_{0},\cdots \;,\hat{x_{i}},\cdots x_{q}\rangle , \end{array}$$

where $\langle x_{0},x_{1},\cdots \;,x_{q}\rangle \in K$ and the $\hat{•}$ denotes the drop-out operation. With the boundary maps, there is a nested relation among chains

$$\begin{array}{l} \cdots \overset{\partial_{n+1}}{\to }C_{n}(K)\overset{\partial_{n}}{\to }C_{n-1}(K)\overset{\partial_{n-1}}{\to }\cdots C_{1}(K)\overset{\partial_{1}}{\to }C_{0}(K). \end{array}$$

This nested relation among chains is known as the *chain complex*, which is denoted as $C={\left\{C_{q},\partial_{q}\right\}}_{q\in \mathbb{Z}}$.

A fundamental result in the homology theory (Munkres, 1993 Lemma 5.3 section 1.5, p. 30) is that the composition of any two consecutive boundary maps is trivial, i.e., ∂_*q*−1_ ° ∂_*q*_ = 0. This result allows one to define the following quotient space. Denote *cycles* and *boundaries* by $Z_{q}\left(K\right)$ and $B_{q}\left(K\right)$, respectively, which are defined as

$$\begin{array}{l} Z_{q}\left(K\right):=\ker\left(\partial_{q}\right)=\left\{c\in C_{q}\;|\;\partial_{q}\left(c\right)=0\right\},B_{q}\left(K\right):=\text{im}\left(\partial_{q+1}\right)\\ \;=\left\{\partial_{q+1}\left(z\right)\in C_{q}\;|\;z\in C_{q+1}\right\}. \end{array}$$

**Figure 3:**
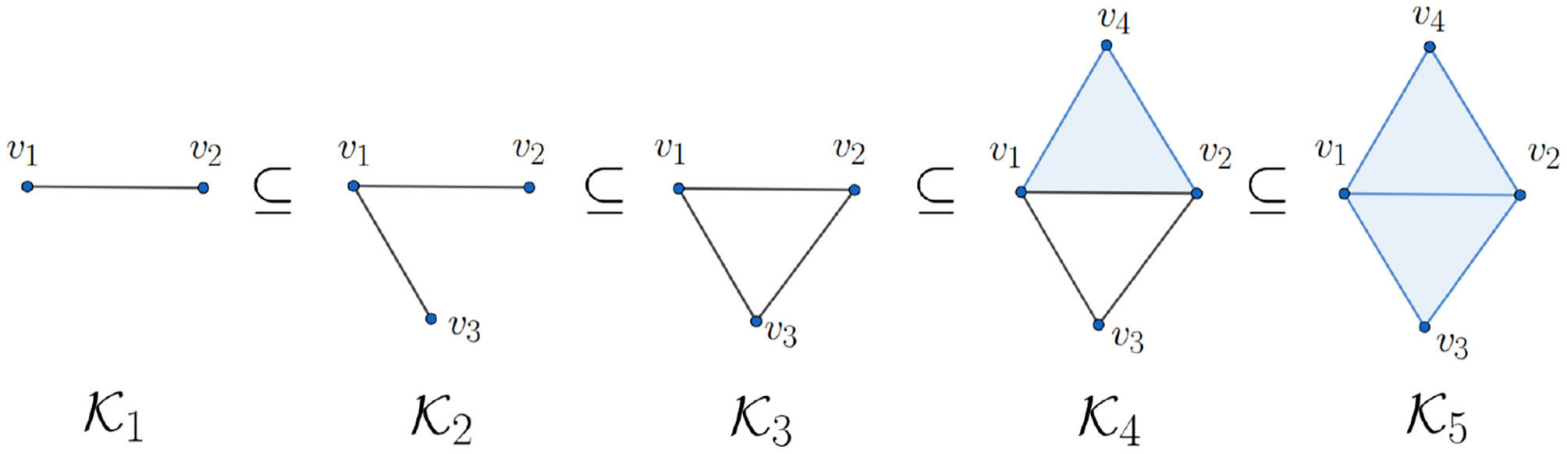
Illustration of a filtration of simplicial complexes $K_{1},K_{2},K_{3},K_{4}$, and $K_{5}$.

Note that each $B_{q}\left(K\right)$ is a subspace of $Z_{q}\left(K\right)$ since ∂_*q*−1_ ° ∂_*q*_ = 0. Therefore, we can define the *q*th *homology* to be the quotient space

(1) $$\begin{array}{l} H_{q}\left(K\right):=\frac{Z_{q}\left(K\right)}{B_{q}\left(K\right)}=\frac{\ker\left(\partial_{q}\right)}{\text{im}\left(\partial_{q+1}\right)}, \end{array}$$

which is again a vector space. For instance, if $K=K_{3}$ in Figure 3, then $H_{0}\left(K_{3}\right)≃{\mathbb{Z}}_{2}$ and $H_{1}\left(K_{3}\right)≃{\mathbb{Z}}_{2}$ because it contains one connected component and one 1-dimensional hole. More precisely, the 1-dimensional hole in $K_{3}$ is represented by the 1-cycle

$$\begin{array}{l} c=\langle v_{1},v_{2}\rangle +\langle v_{2},v_{3}\rangle +\langle v_{3},v_{1}\rangle . \end{array}$$

Actually, a *q*-hole may be represented by different *q*-cycles. For example, if $K=K_{3}$ in Figure 3, then the 1-cycles *c* = 〈*v*_1_, *v*_2_〉 + 〈*v*_2_, *v*_3_〉 + 〈*v*_3_, *v*_1_〉 and *d* = 〈*v*_1_, *v*_4_〉 + 〈*v*_4_, *v*_2_〉 + 〈*v*_2_, *v*_3_〉 + 〈*v*_3_, *v*_1_〉 represent the same 1-dimensional hole in $K_{4}$ because *c* + *d* = 〈*v*_1_, *v*_2_〉 + 〈*v*_1_, *v*_4_〉 + 〈*v*_4_, *v*_2_〉 ∈ im(∂_2_) is the boundary of the 2-simplex 〈*v*_1_, *v*_2_, *v*_4_〉. This gives us an intuition about the algebraic structure (1).

The *q*th *Betti number* is defined to be the dimension of the *q*th homology; that is,

(2) $$\begin{array}{l} \beta_{q}\left(K\right)=\dim\left(H_{q}\left(K\right)\right), \end{array}$$

which measures the number of *q*-dimensional holes. As a result, given a simplicial complex K, the *homology of* K is a collection of vector spaces ${\left\{H_{q}\left(K\right)\right\}}_{q=0}^{\infty }$, and its *Betti numbers* is denoted as $\beta \left(K\right):={\left\{\beta_{q}\left(K\right)\right\}}_{q=0}^{\infty }$.

### 2.3. Persistent Homology

We now introduce a natural generalization of homology, the persistent homology, that is suitable for data analysis. persistent homology is more suitable for data analysis than homology due to this capability of dealing with inevitable noise in real world dataset. It depends on the notion of *filtration* to handle noise. In general, filtration is a sequence of simplicial complexes (see Figure 3 for an example). We are interested in how the “holes” vary in the filtration. Intuitively, if certain holes are “robust,” they will survive in the filtration.

**Definition 1 (Edelsbrunner and Harer, 2010 section 3.4, p. 70)**. *For an index set *I*, a *filtration* is a sequence of simplicial complexes*, ${\left\{K_{t}\right\}}_{t\in I}$, *satisfying*

(3) $$\begin{array}{l} K_{t_{1}}\subseteq K_{t_{2}},whenever\;t_{1}\leq t_{2}. \end{array}$$

From the previous discussion, for each $K_{t}$ in a filtration, one could compute its homology group and Betti number. Because of the nested subset relation in a filtration, there exist relations among simplicial complexes. This allows one to track and record the changes of the homology group and the Betti numbers, which we detail now. Given a fixed *q* ≥ 0, each $K_{i}$ induces homology $H_{q}\left(K_{i}\right)$. Denote $\iota_{i}:K_{i}\to K_{i+1}$ to be the inclusion map. Then $\iota_{i}\left(Z_{q}\left(K_{i}\right)\right)\subseteq Z_{q}\left(K_{i+1}\right)$ and $\iota_{i}\left(B_{q}\left(K_{i}\right)\right)\subseteq B_{q}\left(K_{i+1}\right)$ (Edelsbrunner and Harer, 2010 section 4.4, p. 95). Therefore, the mapping

(4) $$\begin{array}{l} \bar{\iota_{i}}:H_{q}\left(K_{i}\right)\to H_{q}\left(K_{i+1}\right),\;\bar{c}\mapsto \bar{\iota_{i}\left(c\right)} \end{array}$$

induced by ι_*i*_ is a well-defined linear transformation over ℤ_2_. We also define a linear transformation

(5) $$\begin{array}{l} \rho_{q}^{i,i+k}:=\bar{\iota_{i+k-1}}\circ \cdots \circ \bar{\iota_{i+1}}\circ \bar{\iota_{i}}, \end{array}$$

which maps $H_{q}\left(K_{i}\right)$ to $H_{q}\left(K_{i+k}\right)$. The following definition is crucial for defining *lifespans* of connected components or holes in homology theory.

**Definition 2 (Edelsbrunner and Harer, 2010 section 7.1, p. 151)**. *Let* ${\left\{K_{i}\right\}}_{i=0}^{n}$ *be a filtration of simplicial complexes. For* *q* ∈ *ℤ*_≥0_ *and* *i, j* ∈ *ℤ*_≥0_ *with* *i* ≤ *j*, *we define the persistent homology as*

(6) $$\begin{array}{l} H_{q}^{i,j}:=\frac{Z_{q}\left(K_{i}\right)}{B_{q}\left(K_{j}\right)\cap Z_{q}\left(K_{i}\right)}. \end{array}$$

Since $K_{0}\subseteq K_{1}\subseteq \cdots \subseteq K_{n}$, we have inclusions of *q*-chains: $C_{q}\left(K_{0}\right)\subseteq C_{q}\left(K_{1}\right)\subseteq \cdots \subseteq C_{q}\left(K_{n}\right)$ for all *q* ≥ 0. Hence, the intersection $B_{q}\left(K_{j}\right)\cap Z_{q}\left(K_{i}\right)$ is a well-defined subspace of $Z_{q}\left(K_{i}\right)$. Moreover, for *i* ≤ *j*, the kernel of the linear transformation

$$\begin{array}{l} \phi_{q}^{i,j}:Z_{q}\left(K_{i}\right)\to \frac{Z_{q}\left(K_{j}\right)}{B_{q}\left(K_{j}\right)},\;c\mapsto \bar{c}=c+B_{q}\left(K_{j}\right) \end{array}$$

induced by the inclusion map is $B_{q}\left(K_{j}\right)\cap Z_{q}\left(K_{i}\right)$. By the first isomorphism theorem, we obtain an injective linear transformation

$$\begin{array}{l} \bar{\phi_{q}^{i,j}}:\frac{Z_{q}\left(K_{i}\right)}{B_{q}\left(K_{j}\right)\cap Z_{q}\left(K_{i}\right)}\to \frac{Z_{q}\left(K_{j}\right)}{B_{q}\left(K_{j}\right)}. \end{array}$$

Via the one-to-one linear mapping $\bar{\phi_{q}^{i,j}}$, the vector space $H_{q}^{i,j}$ may be viewed as a subspace of $H_{q}\left(K_{j}\right)$. In particular, if *i* = *j*, then $H_{q}^{i,j}=H_{q}\left(K_{i}\right)=H_{q}\left(K_{j}\right)$, which means that the persistent homology is a generalization of the homology. With the inclusion $H_{q}^{i,j}↪H_{q}\left(K_{j}\right)$, we define the moments of *birth* and *death* of a “hole” in the filtration.

**Definition 3 (Edelsbrunner and Harer, 2010 section 7.1, p. 151)**. *Let* ${\left\{K_{i}\right\}}_{i=0}^{n}$ *be a filtration of simplicial complexes and* *q* ∈ *ℤ*_≥0_.

- A *q*-hole $\bar{c}$ ($c\in Z_{q}\left(K_{i}\right)$) is **born** at $K_{i}$ if $\bar{c}\in H_{q}\left(K_{i}\right)\backslash \left\{0\right\}$, but $\bar{c}\notin \text{im}\left(\rho_{q}^{i-1,i}\right)$;
- A *q*-hole $\bar{c}$ ($c\in Z_{q}\left(K_{i}\right)$) **dies** at $K_{j}$ if $\rho_{q}^{i,j-1}\left(\bar{c}\right)\notin H_{q}^{i-1,j-1}$, but $\rho_{q}^{i,j}\left(\bar{c}\right)\in H_{q}^{i-1,j}$.

*The death* *d* = ∞ *means that the *q*-hole never dies in the filtration*.

We use Figure 3 to explain the relation between these two abstract definitions. For instance, the non-trivial element $\bar{c}$ represented by 1-chain *c* = 〈*v*_1_, *v*_2_〉 + 〈*v*_2_, *v*_3_〉 + 〈*v*_3_, *v*_1_〉 in $H_{1}\left(K_{3}\right)$ is born at $K_{3}$ i.e., $\bar{c}\notin \text{im}\left(\rho_{1}^{2,3}\right)$ because $H_{1}\left(K_{2}\right)=\left\{0\right\}$ and ${\mathbb{Z}}_{2}=H_{1}\left(K_{3}\right)={\text{span}}_{{\mathbb{Z}}_{2}}\left\{\bar{c}\right\}$. On the other hand, the fact $\left\{0\right\}\subseteq H_{1}^{2,5}\subseteq H_{1}\left(K_{5}\right)=\left\{0\right\}$ shows that $\rho_{1}^{3,5}\left(\bar{c}\right)\in H_{1}\left(K_{5}\right)=H_{1}^{2,5}$ and $\rho_{1}^{3,4}\left(\bar{c}\right)\notin H_{1}^{2,4}$ because $H_{1}^{2,4}=\left\{0\right\}$ (since $Z_{1}\left(K_{2}\right)=\left\{0\right\}$) and ${\mathbb{Z}}_{2}=H_{1}\left(K_{4}\right)={\text{span}}_{{\mathbb{Z}}_{2}}\left\{\rho_{1}^{3,4}\left(\bar{c}\right)\right\}$, thus $\bar{c}$ dies at $K_{5}$. We refer readers with interest to Edelsbrunner and Harer (2010) for more details in persistent homology.

### 2.4. Persistence Diagram

*Persistence diagram* proposed in Edelsbrunner et al. (2000) or equivalently *persistence barcodes* proposed in Carlsson et al. (2005) is a tool to visualize the complicated lifespans of holes in a given filtration for data analysis. We use persistence diagram in this paper.

The persistence diagram possesses the desired *stability property* (Cohen-Steiner et al., 2007)—a bounded perturbation of a given filtration leads to a bounded perturbation of the corresponding persistence diagram. Due to the inevitable noise in real data, this stability property renders persistence diagram based approaches suitable for data analysis. The bottleneck and Wasserstein distances (Cohen-Steiner et al., 2007) are typical ways to measure differences among persistence diagrams. The formal statements of the stability property based on these two distances are provided in sections 3.1, 3.2. We refer readers with interest to Edelsbrunner and Harer (2010) for details in persistence diagram.

**Definition 4 (Edelsbrunner and Harer, 2010 section 7.1, p. 152)**. *Let* ${\left\{K_{i}\right\}}_{i=0}^{n}$ *be a filtration of simplicial complexes and* *q* ∈ ℤ_≥0_. *The* *qth persistence diagram, denoted as* $P_{q}\left({\left\{K_{i}\right\}}_{i=0}^{n}\right)$, *of the filtration is the multiset of* *q-dimension holes in the filtration*.

In other words, a *q*-dimensional hole in a filtration is recorded by a pair (*b, d*) of integers where *b* and *d* are called the *birth* and *death* of the hole, respectively (Edelsbrunner and Harer, 2010). Although the above definition of persistence diagram seems technical, its interpretation is intuitive. For instance, consider the filtration shown in Figure 3. We look for the “changes” of topological structure (holes). Note that since a connected component is born at $K_{1}$ (specifically, 〈*v*_1_, *v*_2_〉), its birth value is *b* = 1; since it lives throughout the filtration, its death value is ∞. We now turn our focus to the 1-dimensional hole. Note that a 1-dimensional hole (specifically, 〈*v*_1_, *v*_2_〉 + 〈*v*_2_, *v*_3_〉 + 〈*v*_1_, *v*_3_〉) is formed at $K_{3}$, so its birth value is 3; note also that this hole is filled at $K_{5}$, so its death value is 5. Since there is no more change of holes, we have the persistence diagrams $P_{0}\left({\left\{K_{i}\right\}}_{i=1}^{5}\right)=\left\{\left(1,\infty \right)\right\}$ and $P_{1}\left({\left\{K_{i}\right\}}_{i=1}^{5}\right)=\left\{\left(3,5\right)\right\}$.

**Figure 4:**
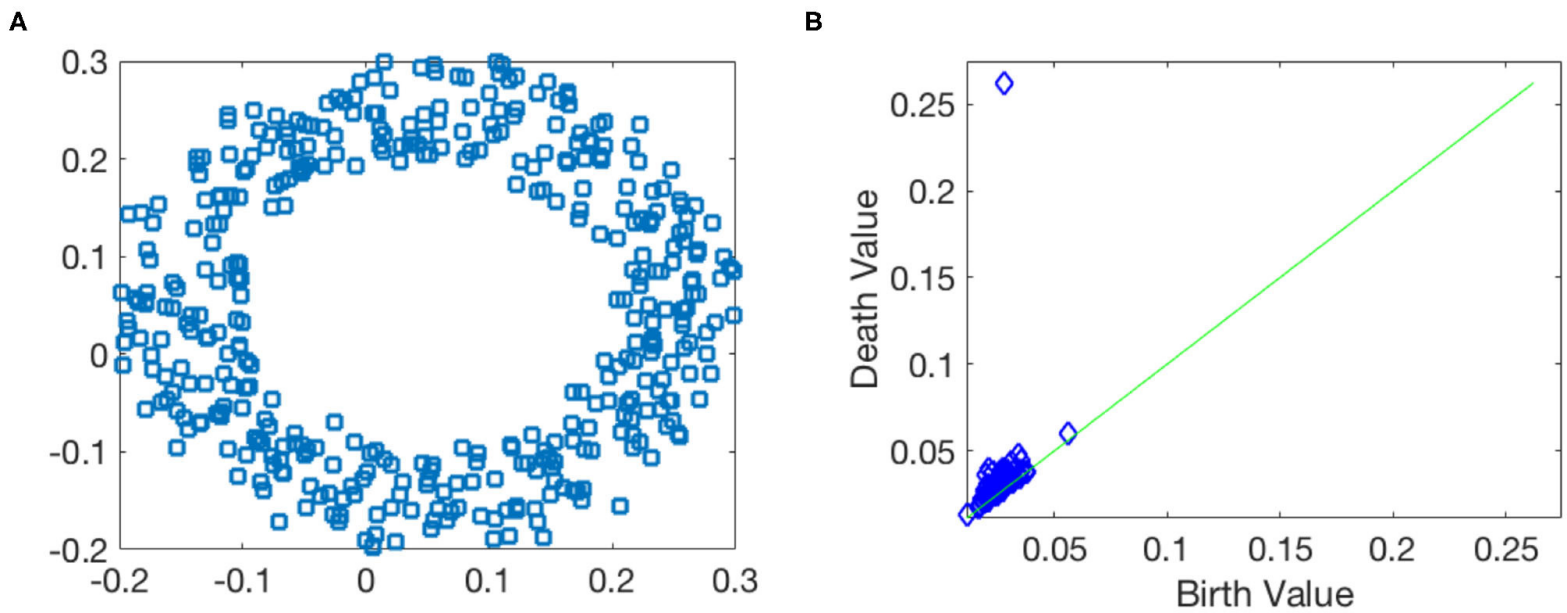
Toy example of the persistent homology. **(A)** Data points are sampled from a circle with the Gaussian noise. **(B)** 1st dimensional persistence diagram.

Before closing this subsection, we illustrate how persistent homology and persistence diagram work by taking a noisy point cloud sampled from a circle contaminated by Gaussian noise shown in Figure 4A. If there is no noise, the 1st Betti number of the circle is β_1_ = 1. In the noisy case, the Betti number information is contained in the form of the persistence diagram as shown in Figure 4B, where each point represents one 1-dimensional hole associated with its birth and death value. In Figure 4B, we observe that there is an outstanding point with long lifespan (located around birth value 0.05 and death value 0.25), while lifespans for other points are very small. This suggests that the noisy point cloud has a strong/robust 1-dimensional hole. This captures the main topology information, β_1_ = 1, about this data.

### 2.5. Data Analysis With Persistence Diagram and Commonly Considered TDA Statistics

Usually, researchers design statistics on the persistence diagram of a given dataset via the chosen filtration. One basic result supporting this approach is Mileyko et al. (2011), where authors showed that the space of persistence diagrams with certain metric is complete and separable. This result forms a theoretic foundation for any statistical methods. In Fasy et al. (2014) and Blumberg et al. (2014), authors derived confidence sets of persistence diagrams in order to separate the long lifespan holes from noisy ones, and also proposed four ways to estimated them. While these theoretical results shed light on applying TDA to analyze complex data, however, any operation in the space of persistence diagrams is complicated and difficult to compute. For example, computing bottleneck or Wasserstein distances among persistence diagrams is a difficult task and can be time consuming, even for the state-of-art algorithm (Kerber et al., 2017). Another result indicates that the mean in the space of persistence diagrams may not be unique (Turner et al., 2014a). This computational burden renders it less applicable to data analysis.

**Figure 5:**
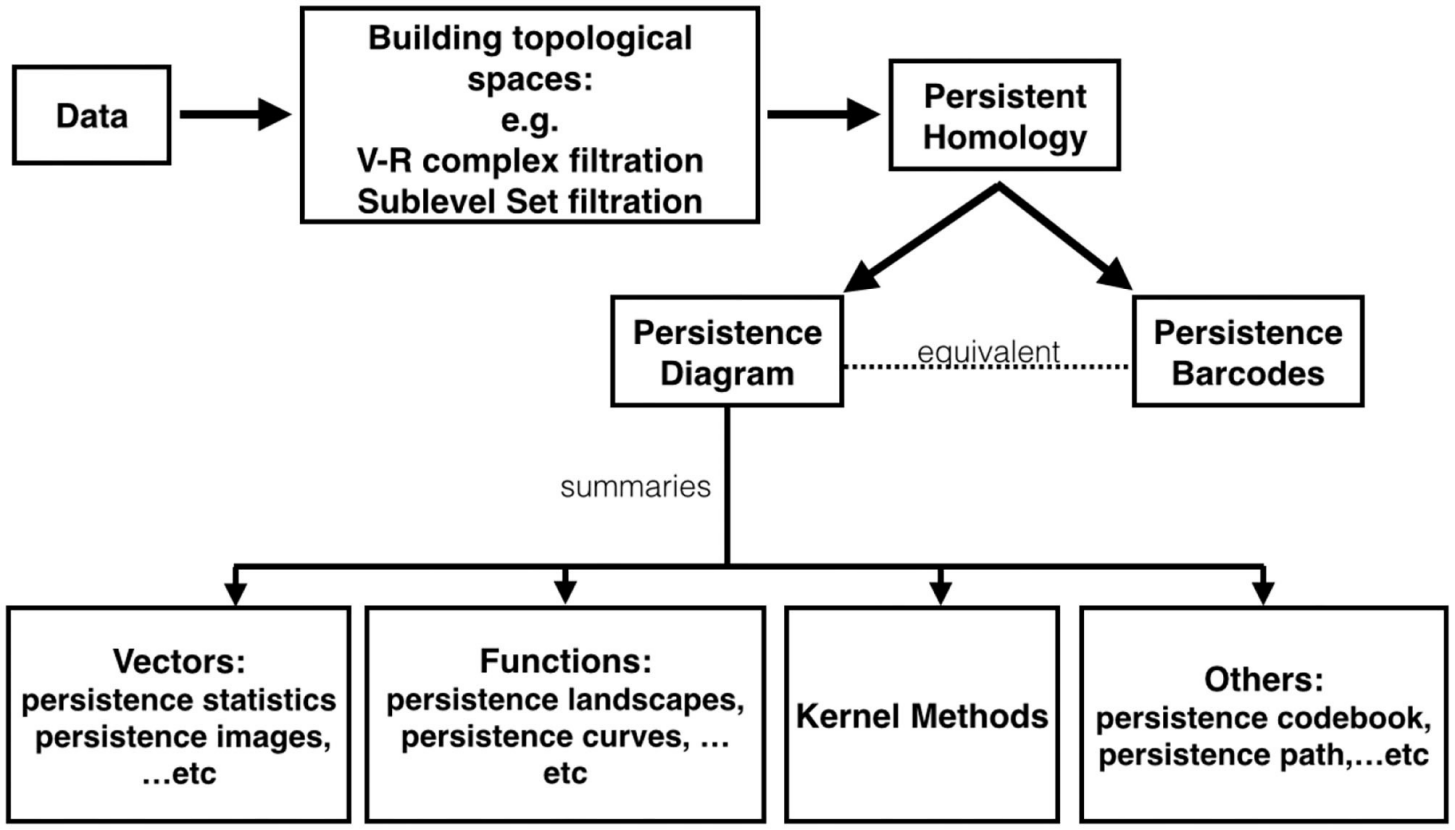
A chart depicting relationship among existing TDA tools.

To get around the computational issue when working with those distances, one major approach is to “vectorize” persistence diagrams; that is, researchers map the space of persistence diagrams into another space. For example, persistence landscapes (Bubenik, 2015) map persistence diagrams into a Banach space, specifically *L*^*p*^ space. More examples include persistence image (Adams et al., 2017), generalized persistence landscapes (Berry et al., 2020), persistence path (Chevyrev et al., 2018), persistence codebook (Zelinski et al., 2020), persistence curves (Chung and Lawson, 2019), kernel based methods (Reininghaus et al., 2015; Kusano et al., 2016), and persistent entropy (Chintakunta et al., 2015; Atienza et al., 2019b). These methods have been studied and applied to different applications. In Figure 5, we provide a chart depicting the relationship among existing TDA tools. We mention that the proposed persistence statistics in section 3 could be viewed as a computationally efficient vectorization of persistence diagrams.

## 3. TDA for Time Series Analysis and Features Extraction

Armed with the theoretical background in section 2, we are ready to describe how to apply TDA for time series analysis. To apply the persistent homology to analyze complicated time series, we introduce two useful filtrations, the sub-level set filtration and the Vietoris-Rips complexes filtration. With these two filtrations, we introduce a novel features extraction methods, coined persistence statistics, based on the persistence diagrams of the sub-level set filtration and the Vietoris-Rips complexes filtration.

### 3.1. First Useful Filtration—Sub-Level Set Filtration

To simplify the discussion and illustrate the idea, we identify a time series as a discretization of a continuous function *f*:[0, *T*] → ℝ, where *T* is some fixed constant. For each *h* ∈ ℝ, the *sub-level set* of *f* is defined as

(7) $$\begin{array}{l} f_{h}:=f^{-1}\left(\left(-\infty ,h\right]\right)=\left\{t\in \left[0,T\right]\;|\;f\left(t\right)\leq h\right\}. \end{array}$$

Clearly, *f*_*h*_1__ ⊆ *f*_*h*_2__ whenever *h*_1_ ≤ *h*_2_. Therefore, for any increasing sequence {*h*_*i*_}_*i*_, the collection of sub-level sets, {*f*_*h*_*i*__}, forms a filtration. Intuitively, the sub-level set filtration reveals the *oscillating information* of the functions. Since each *f*_*h*_ is a subset of [0, *T*] ⊆ ℝ, it only contains 0-dimensional structures, i.e., connected components. Hence, the only non-trivial persistence diagram in this case is $P_{0}$. For simplicity, when there is no danger of confusion, for a given function *f*, we use $P_{0}\left(f\right)$ to denote $P_{0}\left(\left\{f_{h_{i}}\right\}\right)$, the persistence diagram associated with the sub-level sets filtration of *f*. As discussed in Edelsbrunner and Harer (2010), each element in $P_{0}$ is a min-max pair in the original function *f*(*t*). The concept of this filtration is closely related to the size function theory (see Biasotti et al., 2008 and references therein) and is commonly used as a shape descriptor (Biasotti et al., 2008). In practice, persistence diagram is robust to noise under the *bottleneck distance*. This fact renders persistence diagram an useful data analysis quantity. A precise statement of this *robustness* is below.

**Theorem 3.1.1**. *Let *X* be an *n*-dimensional rectangle in* ℝ^*n*^. *Take two continuous functions* *f, g* : *X* → ℝ *with finitely many local extremums (minimums or maximums). Then, we have for* *q* ∈ ℕ,

$$\begin{array}{l} d_{B}\left(P_{q}\left(f\right),P_{q}\left(g\right)\right)\leq ∥f-g{∥}_{\infty }, \end{array}$$

*where* *d*_*B*_ *is the* bottleneck distance *defined as* $d_{B}\left(P_{q}\left(f\right),P_{q}\left(g\right)\right)=\inf_{\gamma }\sup_{\left(b,d\right)\in P_{q}\left(f\right)}∥\left(b,d\right)-\gamma \left(b,d\right){∥}_{\infty }$, *where γ ranges over all bijections from* $P_{q}\left(f\right)$ *to* $P_{q}\left(g\right)$ *considering the infinite points on the diagonal*.

In fact, Theorem 3.1.1 is a special form of a stability theorem (Main Theorem in Cohen-Steiner et al., 2007, p. 109). See Supplementary Material (section 1.3) for an illustrative example of the sub-level sets filtration.

### 3.2. Second Useful Filtration—Vietoris-Rips Complexes Filtration

To introduce Vietoris-Rips (VR) complexes filtration for a given time series, we first embed the time series into a high dimension point cloud via *Taken's lag map* (Takens, 1981), which is constructed in the following way. Take *p* ∈ ℕ to be the dimension of the embedding, and τ ∈ ℕ to be the lag step. For a given time series **x** : ℤ → ℝ, the lag map with lag τ and dimension *p* is defined as

(8) $$\begin{array}{l} R_{p,\tau }\left(\text{x}\right)=\left\{{\left(\text{x}\left(t\right),\text{x}\left(t-\tau \right),\text{x}\left(t-2\tau \right),\ldots ,\text{x}\left(t-\left(p-1\right)\tau \right)\right)}^{⊤}|t\in \mathbb{Z}\right\}, \end{array}$$

which is a subset of ℝ^*p*^. We postpone details of Taken's lag map to Supplementary Material (section 1.2). With the point cloud $R_{p,\tau }\left(\text{x}\right)\subseteq {\mathbb{R}}^{p}$, we are ready to introduce the Vietoris-Rips complex.

In general, given a point cloud $X=\left\{x_{1},\ldots ,x_{N}\right\}\subset {\mathbb{R}}^{p}$, the main idea of Vietoris-Rips complex is to build simplicial complexes from X if points in X are closed enough. A formal definition is given below.

**Definition 5 (Edelsbrunner and Harer, 2010 section 3.2, p. 61)**. *Let* $X=\left\{x_{1},x_{2},\ldots ,x_{N}\right\}\subseteq {\mathbb{R}}^{p}$ *be a point cloud and take* ϵ > 0. *The Vietoris-Rips complex is a collection of all simplexes σ with vertices in* X *with* diam(σ) ≤ 2ϵ, *where* diam(σ) *is the diameter of σ; that is*,

(9) $$\begin{array}{l} \text{VR}\left(X;\epsilon \right):=\overset{p}{\underset{q=0}{⋃}}\left\{q-\text{simplex }\sigma \;|\text{ diam}\left(\sigma \right)\leq 2\epsilon ,\text{Vert}\left(\sigma \right)\subseteq X\right\}. \end{array}$$

Clearly, for an increasing sequence ϵ_1_ < ϵ_2_ < ⋯ < ϵ_*N*_, the corresponding sequence of Vietoris-Rips complexes forms a filtration:

(10) $$\begin{array}{l} \text{VR}\left(X;\epsilon_{1}\right)\subseteq \text{VR}\left(X;\epsilon_{2}\right)\subseteq \cdots \subseteq \text{VR}\left(X;\epsilon_{N}\right). \end{array}$$

After determining the representation rules of connected components, the lifespan of holes of different dimensions can be computed easily. See Supplementary Material (section 1.3) for an illustrative example of the Vietoris-Rips filtration.

For simplicity, we denote the *q*-th persistence diagram associated with the Vietoris-Rips filtration as $P_{q}\left(R_{p,\tau }\left(\text{x}\right)\right):=P_{q}\left({\left\{\text{VR}\left(R_{p,\tau }\left(\text{x}\right);\;\epsilon \right)\right\}}_{\epsilon }\right)$. In parallel with Theorem 3.1.1, the stability of persistence diagrams extracted from a Vietoris-Rips filtration has been discussed in Chazal et al. (2014).

**Theorem 3.2.1 (Chazal et al., 2014, Theorem 5.2)**. *For finite metric spaces* (*X, d*_*X*_) *and* (*Y, d*_*Y*_), *then for* *q* ∈ ℕ,

$$\begin{array}{l} d_{B}\left(P_{q}\left(\text{VR}\left(X\right)\right),P_{q}\left(\text{VR}\left(Y\right)\right)\right)\leq 2d_{GH}\left(X,Y\right), \end{array}$$

*where* *d*_*B*_ *is the* bottleneck distance *and* *d*_*GH*_ *is the Gromov-Hausdorff distance*.

The formal definition of Gromov-Hausdorff distance can be found in Chazal et al. (2014) and Burago et al. (2001), and conceptually, it measures the similarity between two metric spaces under distance-preserving transformations.

### 3.3. Persistence Statistics

We now introduce a set of new features to summarize persistence diagrams. It is computationally efficient and straightforward to implement. We propose to explore distributions of the birth *b* and the death *d* of all possible holes, and calculate their statistic measurements. This idea is considered one of the most straightforward way to extract features from persistence diagrams (Pun et al., 2018). Despite its simplicity, it has been used in several studies, such as skin lesions classification (Chung et al., 2018), bifurcations analysis in dynamical systems (Mittal and Gupta, 2017), and protein classification (Cang et al., 2015).

To be more specific, given a persistence diagram P, we transform it into two multi-sets of numbers, *M* and *L*, defined as

(11) $$\begin{array}{l} M=\left\{\frac{d+b}{2}\;|\;\left(b,d\right)\in P\right\}\text{ and }L=\left\{d-b\;|\;\left(b,d\right)\in P\right\}. \end{array}$$

Note that for the Vietoris-Rips complex filtration, $\frac{d+b}{2}$ captures the “size” of the associated hole, and *d* − *b* captures the robustness of the associated hole. On the other hand, for the sublevel set filtration, $\frac{d+b}{2}$ reveals the locations of holes, and *d* − *b* captures the differences between low and high peaks in a time series. Note that since the hole (0, ∞) always exists in the persistence diagram as is shown in the previous section, it is omitted.

In this paper, for each persistence diagram, we consider eight summary statistics to represent the multi-set *M*, including mean, standard deviation, skewness, kurtosis, 25th, 50th, 75th percentile, and the *persistent entropy* (Chintakunta et al., 2015). We number them from 1 to 8. We consider the same summary statistics for the multi-set *L*, and number them from 9 to 16.

**Definition 6 (Persistence Statistics)**. *Given a persistence diagram, the persistence statistics (PS) is defined as a map*, Φ^(*PS*)^, *that transforms the persistence diagram to a point in* ℝ^16^.

As shown in Algorithm 1, for the Vietoris-Rips complex filtration, we consider 0-th and 1-th persistence diagrams; for the sub-level set filtration, we consider 0-th persistence diagram.

The persistent entropy of *M* and *L*, denoted as *E*(*M*) (Chung and Lawson, 2019) and *E*(*L*) (Atienza et al., 2020) respectively, describes the complexity of *M* and *L*. They are formally defined by

$$\begin{array}{l} E\left(M\right)=\underset{m\in M}{\sum }\left[-\frac{m}{\underset{m^{\prime }\in M}{\sum }m^{\prime }}\log\frac{m}{\underset{m^{\prime }\in M}{\sum }m^{\prime }}\right]\text{ and }E\left(L\right)\\ \;=\underset{l\in L}{\sum }\left[-\frac{l}{\underset{l^{\prime }\in L}{\sum }l^{\prime }}\log\frac{l}{\underset{l^{\prime }\in L}{\sum }l^{\prime }}\right]. \end{array}$$

*E*(*L*) has been used to study the cell arrangements (Atienza et al., 2019a), emotion recognition (Gonzalez-Diaz et al., 2019), and epileptic seizures detection in EEG signals (Piangerelli et al., 2018). From the theoretical perspective, *E*(*L*) is a stable measurement (Theorem 3.12 in Atienza et al., 2020). *E*(*M*) was first appeared in Chung et al. (2018) and the discussion about the stability of *E*(*M*) can be found in Chung and Lawson (2019).

Note that while *intuitively*, holes with long lifespans are considered important features and those with short lifespans are considered noises, in our proposed features, we do not discriminate holes with long or short lifespans. In other words, we take all holes into consideration. This approach is supported by a recent discovery that those considered as noisy holes might actually contain important information. For example, in the drivers' behavior classification (Bendich et al., 2016), authors transformed the space of persistence diagrams into “binned” diagrams, and found that the main differences occurred in those short lifespan holes. Another work on the leave classification (Patrangenaru et al., 2018) also suggested that holes with short lifespans could better distinguish different types of leaves.

## 4. Application to Sleep Stage Classification

In recent decades, a growing body of evidence shows that sleep is not only intimately related to personal health (Karni et al., 1994; Kang et al., 2009) but also has a direct impact on public health (Colten and Altevogt, 2006). In clinics, sleep experts score sleep stage by reading the electroencephalogram (EEG), electrooculogram (EOG), and electromyogram (EMG) based on the American Academy of Sleep Medicine (AASM) criteria (Iber et al., 2007; Berry et al., 2012). Sleep, however, impacts the whole body, and we can read sleep via reading physiological signals other than EEG, particularly ECG and HRV mentioned in section 1. The relationship between HRV and sleep dynamics has been widely studied in the physiology society (Zemaityte and Varoneckas, 1984; Vaughn et al., 1995; Toscani et al., 1996; Bonnet and Arand, 1997; Elsenbruch et al., 1999; Chouchou and Desseilles, 2014; Penzel et al., 2016). Specifically, when a subject is awake, since the sympathetic tone of the ANS is dominant, he/she has a higher heart rate and a less stable heart rhythm due to external stimuli (Somers et al., 1993). When a subject is asleep, the heart rate is lower, and it reaches its lowest value during deep (slow wave) sleep (Snyder et al., 1964). During NREM (non-rapid eye movement) sleep, the parasympathetic nervous system dominates the sympathetic tone and the energy restoration and metabolic rates reach their lowest levels, so the heart rate decreases and the rhythm of the heart stabilizes (Somers et al., 1993).

The above physiological facts indicate that the heart rate rhythm provides a non-invasive window for researchers to study sleep. There have been several studies trying to classify sleep stages based *solely* on HRV. Most of them focus on classifying wake and sleep (Lewicke et al., 2008; Long et al., 2012; Aktaruzzaman et al., 2015; Ye et al., 2016; Malik et al., 2018), some focus on detecting drowsiness (Vicente et al., 2016), and some focus on classifying rapid eye movement (REM) and NREM (Mendez and Matteucci, 2010), or wake, REM, and NREM (Xiao et al., 2013). The challenge and difficulty of this mission can be appreciated from the reported results. In this section, we apply the TDA tool and the proposed persistence statistics to study this problem.

### 4.1. Datasets

The databases we use here are the same as those used in Malik et al. (2018). Here we summarize them and refer readers with interest in the database details to Malik et al. (2018). The *CGMH-training* database consists of standard polysomnogram (PSG) signals on patients suspicious of sleep apnea syndrome at the sleep center in Chang Gung Memorial Hospital (CGMH), Linkou, Taoyuan, Taiwan. The Institutional Review Board of CGMH approved the study protocol (No. 101-4968A3). All recordings were acquired on the Alice 5 data acquisition system (Philips Respironics, Murrysville, PA). Each recording lasts for at least 5 h. The sleep stages, including wake, REM, and NREM (REM and NREM constitute the *sleep* stage), were annotated by two experienced sleep specialists according to the AASM 2007 guidelines (Iber et al., 2007), and a consensus was reached. According to the protocol, the sleep specialists provide annotation for non-overlapping 30 s long epochs. In this study, we focus on the second lead of the ECG recording, which was sampled at 200 Hz. There are 90 participants without sleep apnea [each with apnea-hypopnea index (AHI) <5] in this database, among which we consider only 56 participants who have at least 10% epochs labeled as wake to avoid the imbalanced data issue.

We consider three validation databases. The first one is the *CGMH-validation database*. This database consists of 27 participants acquired independently of CGMH-training from the same sleep laboratory in CGMH under the same Institutional Review Board. The other two validation databases are publicly available. The DREAMS Subjects Database^1^ (DREAMS), consists of 20 recordings from healthy participants, where the ECG recordings were acquired by the Brainnet™ system (Medatec, Brussels, Belgium). The sampling rate is 200 Hz, and the minimum recording duration is 7 h. Although the race information is not provided, we may assume that its population constitution is different from that of the CGMH databases since it is collected from Belgium. This database is chosen to assess the model's performance on participants of a different race recorded from different recording machine. The third database is the St. Vincent's University Hospital/University College Dublin Sleep Apnea Database (UCDSADB) from Physionet (Goldberger et al., 2000)^2^. It consists of 25 participants with sleep apnea of various severities. The ECG signal was recorded by Holter monitor at the sampling rate of 128 Hz. The minimum recording is 6 h long. We focus on the first ECG lead in this study. The UCDSADB is chosen to assess the model's performance on recordings which come from participants with sleep disorders. We remark the these validation databases are not used to tune the model's parameters, and no subject is rejected.

### 4.2. Time Series to Analyze—Instantaneous Heart Rate

The data preprocessing steps are the same as those shown in Malik et al. (2018). Here we summarize those steps and refer readers to Malik et al. (2018) for more details. First, apply a standard automatic R peak detection algorithm (Elgendi, 2013). Suppose there are *n*_*k*_ R peaks in the *k*-th subject's ECG recording. Denote ${\left\{r_{k,i}\right\}}_{i=1}^{n_{k}}$ the location in time (sec) of the detected R peaks of the *k*-th subject. We apply the 5-beat median filter to remove artifacts in the detected R peaks; that is, if a detected beat is too close or too far from their preceding beats, it is removed or interpolated. Then, the IHR of the *k*-th recording, denoted as **x**_*k*_, is determined by the shape-preserving piecewise cubic interpolation (Task Force of the European Society of Cardiology and others, 1996) over the nonuniform sampling

(12) $$\begin{array}{l} {\text{x}}_{k}\left(r_{k,i}\right)=60{\left(r_{k,i}-r_{k,i-1}\right)}^{-1}. \end{array}$$

**x**_*k*_ describes the IHR at each time in beats-per-minute. The IHR is sampled at a sampling rate of 4 Hz. We break the IHR signal into 30-s epochs following the same epoch segmentation in the experts' annotations. We discard all epochs with fewer than 5 detected R peaks. This step is adjusted by physiological knowledge. For each labeled epoch, we build a time series of 90 s in length by concatenating the epoch with the preceding 2 epochs. For the sake of handling the inter-individual variance, each 90 s time series is normalized by subtracting its median value. Thus, for the *j*-th epoch of the *k*-th recording, the associated time series we consider is

(13) $$\begin{array}{l} {\text{x}}^{\left(k,j\right)}:=[{\text{x}}_{k}\left(t_{j}-359/4\right),{\text{x}}_{k}\left(t_{j}-358/4\right),\ldots ,{\text{x}}_{k}\left(t_{j}-1/4\right),{\text{x}}_{k}\left(t_{j}\right){]}^{⊤}\\ \;-\text{median}\left\{{\text{x}}_{k}\left(t_{j}-\left(q-1\right)/4\right)|q=1,\ldots ,360\right\}\in {\mathbb{R}}^{360}, \end{array}$$

where *t*_*j*_ indicates the ending time of the *j*-th epoch.

### 4.3. IHR Time Series and Their Persistence Diagrams

Following the discussion in section 3, we apply TDA to IHR time series defined in (13), **x**^(*k, j*)^. More precisely, we consider $P_{0}\left({\text{x}}^{\left(k,j\right)}\right)$ via the sub-level set filtration, and $P_{i}\left(R_{120,1}\left({\text{x}}^{\left(k,j\right)}\right)\right)$ for *i* = 0, 1, via the Vietoris-Rips complex filtration. We extract persistence statistics from both $P_{0}\left({\text{x}}^{\left(k,j\right)}\right)$ and $P_{i}\left(R_{120,1}\left({\text{x}}^{\left(k,j\right)}\right)\right)$, where *i* = 0, 1. We summarize section 3 and highlight our approach in the following pseudocode. See also Figure 2 for an illustration.

**Algorithm 1:** Feature Extraction Scheme

**Input:** A time series, **x**(*t*).
**Output:** Topological features used in this article.
1. Calculate $P_{0}\left(\text{x}\right)$ via sublevel set filtration (as in section 3.1).
2. Calculate $P_{i}\left(R_{120,1}\left(\text{x}\right)\right)$ via VR complex filtration (as in section 3.2).
3. Calculate PS features: $[\Phi^{\left(\text{PS}\right)}\left(P_{0}\left(\text{x}\right)\right),\;\Phi^{\left(\text{PS}\right)}\left(P_{0}\left(R_{120,1}\left(\text{x}\right)\right)\right),\;\Phi^{\left(\text{PS}\right)}\left(P_{1}\left(R_{120,1}\left(\text{x}\right)\right)\right)]$ (as in section 3.3).

**Figure 6:**
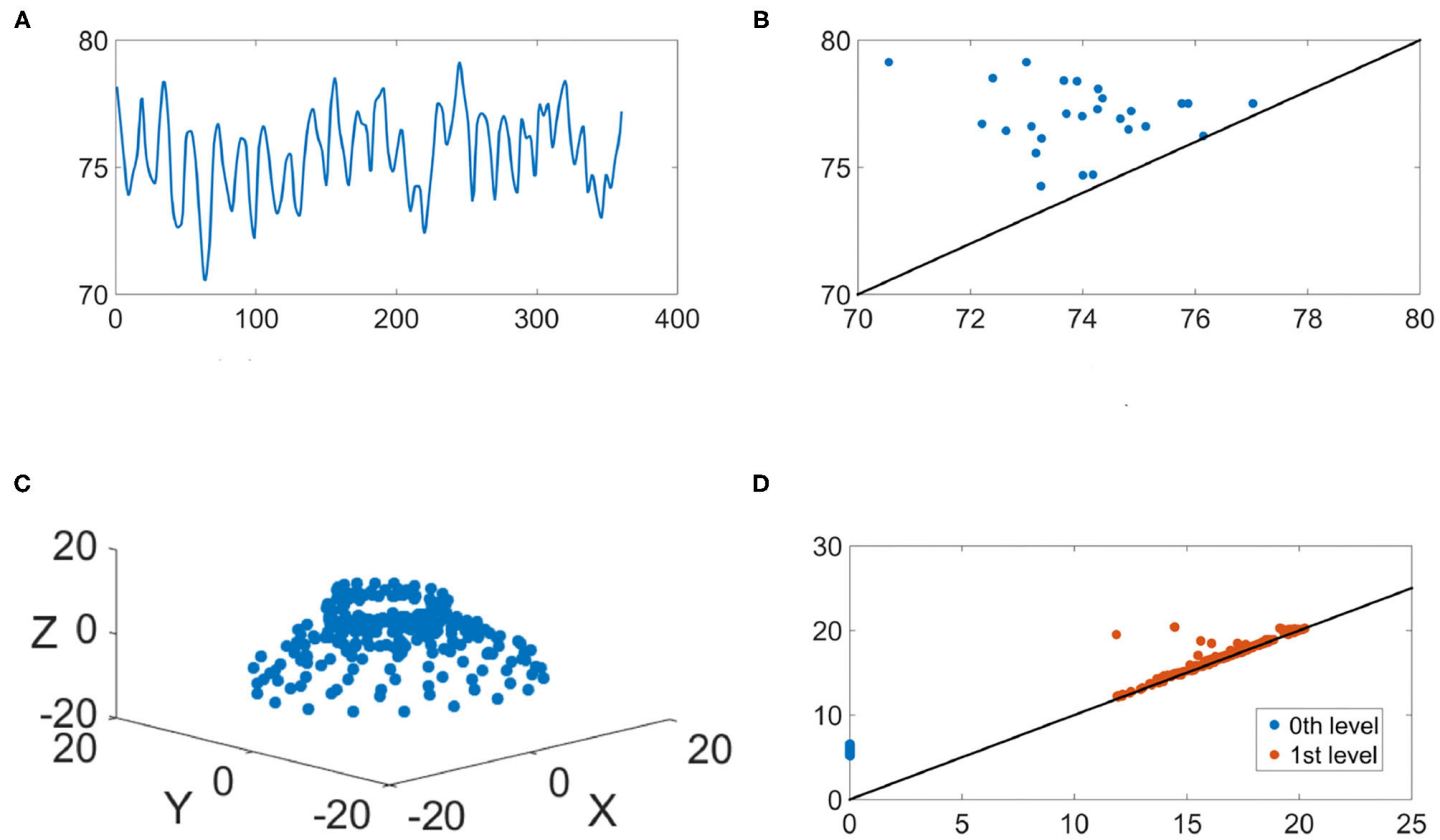
An illustration of the IHR during the wake stage. **(A)** The IHR signal **x**^(*k, j*)^; **(B)** the persistence diagram of the sub-level set filtration, $P_{0}\left({\text{x}}^{\left(k,j\right)}\right)$; **(C)** The first three principal components of the point cloud $R_{120,1}\left({\text{x}}^{\left(k,j\right)}\right)$; **(D)** the persistence diagrams of the Vietoris-Rips filtration, $P_{0}\left(R_{120,1}\left({\text{x}}^{\left(k,j\right)}\right)\right)$ and $P_{1}\left(R_{120,1}\left({\text{x}}^{\left(k,j\right)}\right)\right)$ are superimposed, where blue and red points represent *q* = 0 and *q* = 1, respectively.

**Figure 7:**
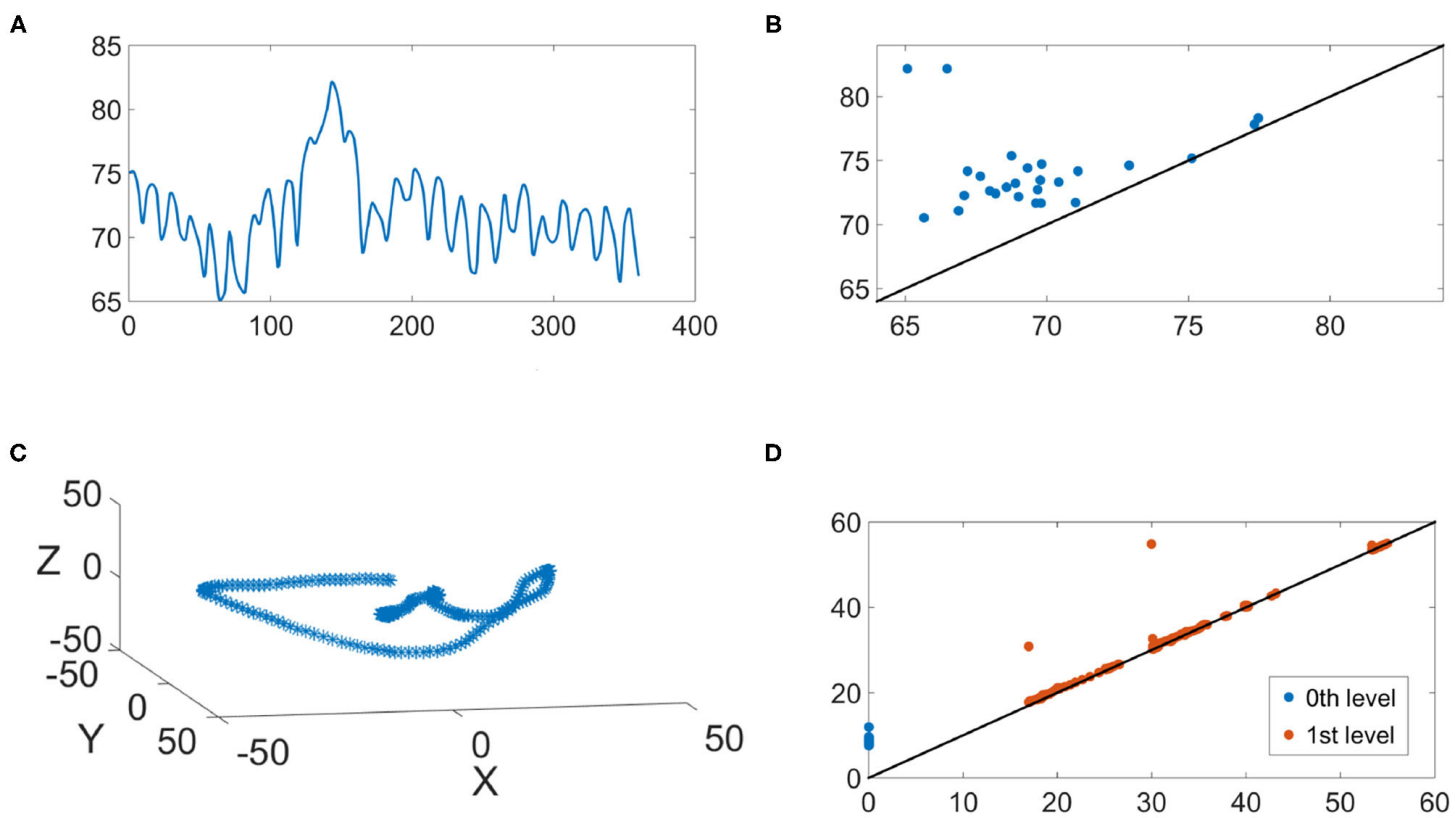
An illustration of the IHR during the sleep stage. **(A)** The IHR signal **x**^(*k, j*)^; **(B)** The persistence diagram of the sub-level set filtration, $P_{0}\left({\text{x}}^{\left(k,j\right)}\right)$; **(C)** The first three principal components of the point cloud $R_{120,1}\left({\text{x}}^{\left(k,j\right)}\right)$; **(D)** the persistence diagrams of the Vietoris-Rips filtration $P_{0}\left(R_{120,1}\left({\text{x}}^{\left(k,j\right)}\right)\right)$ and $P_{1}\left(R_{120,1}\left({\text{x}}^{\left(k,j\right)}\right)\right)$ are superimposed, where blue and red points represent *q* = 0 and *q* = 1, respectively.

We illustrate the IHR time series and their persistence diagrams with different filtrations in Figures 6, 7. From a IHR time series during a wake (resp. sleep) epoch shown in Figure 6A (resp. Figure 7), we observe that these IHR's seem to be different: wake epoch seems to have more variability than sleep one does. Sub-level set filtration captures such variability in the form of the persistence diagram. As shown in Figures 6B, 7B, their persistence diagram's of sub-level set filtration are different. Points in Figure 6B spread widely while most points in Figure 7B are clustered around lower left portion of the diagram. Moreover, Figure 6B seems to have more long-lived points than Figure 7B does. Next, we examine the persistence diagrams of Vietoris-Rips complex filtration. In this work, we take (*p*, τ) = (120, 1), where *p* = 120 is equivalent to a 30 s long time series (since the sampling rate is 4 Hz). This set of parameters is motivated/guided by the AASM criteria where the sleep stage is assigned based on the 30-s readings. The parameters (120, 1) can be thought as sliding a window of a 30-s long time series, and $P\left(R_{120,1}\left(\text{x}\right)\right)$ stores information about changes of this 30-s sliding window over time. Figures 6C, 7C show examples of $R_{120,1}\left({\text{x}}^{\left(k,j\right)}\right)$ projected onto their first three principal components. Visually, the point clouds of Figures 6C, 7C have different shapes (former seems to have a “lamp” shape while the latter does not), and their persistence diagrams shown in Figures 6D, 7D are also different. For instance, the red points in Figure 6D cluster around birth values 10 ~ 20, the red points in Figure 7D have three clusters around the birth values 15 ~ 25, 30 ~ 40, and 55. It is important to note that the computations on $P_{0}\left(R_{120,1}\left({\text{x}}^{\left(k,j\right)}\right)\right)$ are done on the ℝ^120^ space, and projection onto their first three principal components is merely for the visualization purpose.

**Figure 8:**
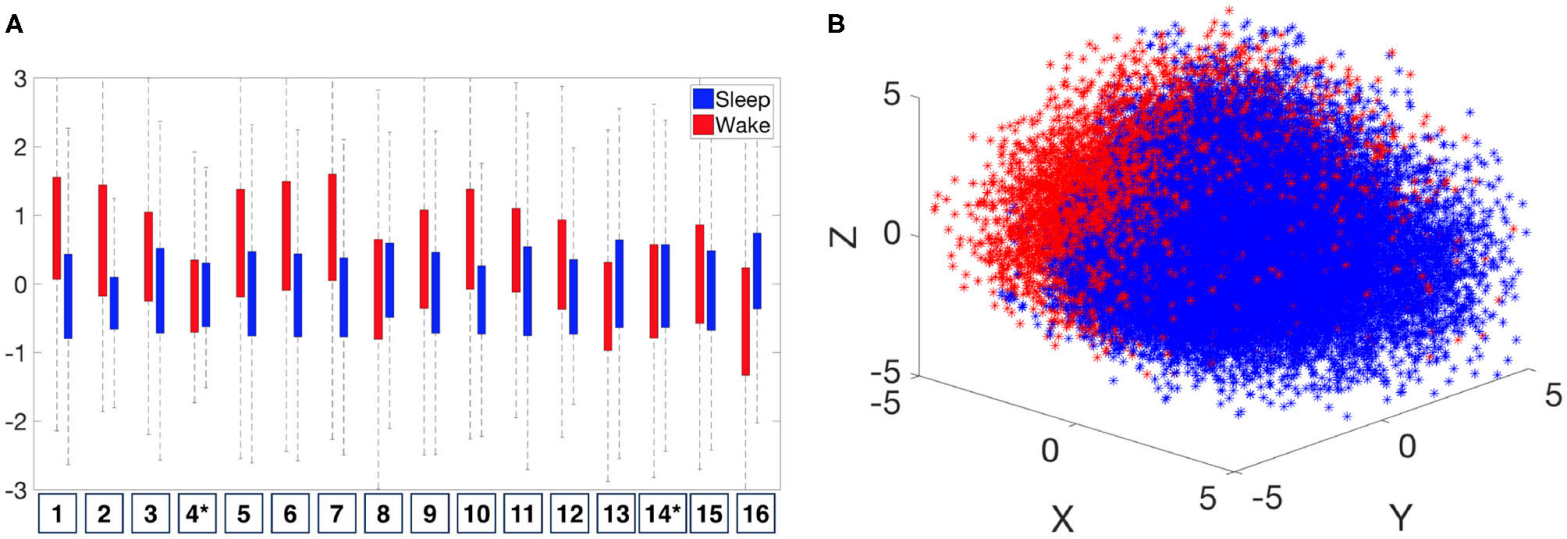
Distribution of normalized persistence statistics features, $\Phi^{\left(\text{PS}\right)}\left(P_{0}\left({\text{x}}^{\left(k,j\right)}\right)\right)$. **(A)** Boxplot of the $\Phi^{\left(\text{PS}\right)}\left(P_{0}\left({\text{x}}^{\left(k,j\right)}\right)\right).$ The numbers listed on the horizontal axis indicates the number of persistence statistics. *Indicates that the feature *fails* to reject the null hypothesis (that two samples have equal medians) of the significance level of 0.05 on the rank sum test with Bonferroni correction. **(B)** Visualization of ${⋃}_{k}{\left\{\Phi^{\left(\text{PS}\right)}\left(P_{0}\left({\text{x}}^{\left(k,j\right)}\right)\right)\right\}}_{j=1}^{n_{k}}$ by the first three principal components.

As discussed in section 2.4, while it is possible to analyze the data via persistence diagrams, it is usually computationally challenging. The proposed persistence statistics allows us to further summarize the persistence diagrams and quantify the above observations. To examine the persistence statistics features, take ${⋃}_{k}{\left\{\Phi^{\left(\text{PS}\right)}\left(P_{0}\left({\text{x}}^{\left(k,j\right)}\right)\right)\right\}}_{j=1}^{n_{k}}$ as an example. In order to compare them on the same scale, we perform the standard *z*-score normalization for each subject. We abuse the notation and use $\Phi^{\left(\text{PS}\right)}\left(P_{0}\left({\text{x}}^{\left(k,j\right)}\right)\right)$ to denote the normalized parameters. In Figure 8A, we show the boxplot of each normalized persistence statistics parameter, where blue (red) bars represent the persistence statistics associated with an IHR time series associated with the sleep (wake) stage. We performed a rank sum test with the null hypothesis that two samples have equal medians with a significance level of 0.05 with the Bonferroni correction. We found that there are significant differences between waking and sleeping features for all persistence statistics parameters, except for the kurtosis of *M* (labeled as 4 in Figure 8A), and the median of *L* (labeled as 14 in Figure 8A). The boxplot as shown in Figure 8A shows that the mean and standard deviation of *M* are the most distinguishable persistence statistics parameters between sleep and wake epochs. To further visualize these features, we apply the principle component analysis (PCA) to ${⋃}_{k}{\left\{\Phi^{\left(\text{PS}\right)}\left(P_{0}\left({\text{x}}^{\left(k,j\right)}\right)\right)\right\}}_{j=1}^{n_{k}}$, and plot the first three principal components in ℝ^3^ as shown Figure 8B. We observe a separation between sleep and wake features. The visualization of $\Phi^{\left(\text{PS}\right)}\left(P_{i}\left(R_{120,1}\left({\text{x}}^{\left(k,j\right)}\right)\right)\right)$, where *i* = 0, 1, is shown in Supplementary Figure 5.

Motivated by the above observation and discussion, we consider the following features for **x**^(*k, j*)^ to distinguish sleep and wake epochs:

(14) $$\begin{array}{l} {}[\Phi^{\left(\text{PS}\right)}(P_{0}(x^{\left(k,j\right)})),\;\Phi^{\left(\text{PS}\right)}(P_{0}(R_{120,1}(x^{\left(k,j\right)}))),\\ \Phi^{\left(\text{PS}\right)}(P_{1}(R_{120,1}(x^{\left(k,j\right)})))]. \end{array}$$

### 4.4. Automatic Sleep Stage Annotation System

#### 4.4.1. Support Vector Machine as the Learning Model

We consider the widely applied classifier with a solid theoretical foundation, the support vector machine (SVM), to establish the heartbeat classification model. This is Step 4 (machine learning step) shown in Figure 2. The linear function kernel is considered in this work and we use the Matlab built-in function fitcsvm with default parameters. The input data are features shown in (14), which are calculated by the publicly available libraries DIPHA (https://github.com/DIPHA/dipha) and Ripser (https://github.com/Ripser/ripser). When there are more than 2 classes, we apply the Error-Correcting Output Codes (ECOC) Dietterich and Bakiri (1994) with one-versus-one design. Specifically, we use the Matlab built-in function fitcecoc with default parameters. The Matlab version is 2019b.

#### 4.4.2. Statistics

We carry out the *cross-database validation*. Specifically, we train the SVM model on one database, and evaluate the performance on the other databases. One of the main challenges in this automatic annotation problem is that the datasets are usually imbalanced; for example, the number of wake epochs is usually much smaller than that of sleep epochs (e.g., in the CGMH-training, the total number of wake epochs is 9, 150, while the total number of sleep epochs is 54, 547). Learning on imbalanced datasets is one of challenging topics in machine learning (He and Ma, 2013; Kuhn and Johnson, 2013; Fernández et al., 2018). Typically, the accuracy would heavily skew toward the majority case. Taking the above CGMH-training as an example, if a model predicts all epochs as sleep, then its accuracy is 54547/(9150 + 54547) ≈ 85%, which may seem high at the first glance. However, this model is clearly useless because it has no predictability for the wake, which can be seen through the sensitivity, 0/9150 = 0. Therefore, in the case of imbalanced datasets, both sensitivity and specificity would be important indicators to evaluate the performance of a model. They both should be as high as possible, and they should be on the similar level.

In order to account for the imbalanced dataset, we adopt a down-sampling process. Let *E*_*s*_ and *E*_*w*_ be the collection of all sleep and wake epochs, respectively, across all subjects in the training set, and denote their cardinality by |*E*_*s*_| and |*E*_*w*_|, respectively. We take all epochs in *E*_*w*_, and randomly select |*E*_*w*_| epochs from *E*_*s*_. The SVM model will then be built on these balanced epochs. Once the model is built on the training dataset, we test it on the entire testing dataset.

We report the following performance measurement indices. When there are *m* labels, denote *M* ∈ ℝ^*m* × *m*^ to be the confusion matrix of the automatic classification model, where *M*_*kl*_ represents the count of epochs whose known group labels are *k* and whose predicted group labels are *l*. The sensitivity (SE), positive predictivity (+P) and F1 for the *k*-th class, the Cohen kappa, and the overall accuracy (Acc) are defined as

(15) $$\begin{array}{l} SE_{k}=\frac{M_{kk}}{\sum_{l=1}^{m}M_{kl}},\;+P_{k}=\frac{M_{kk}}{\sum_{l=1}^{m}M_{lk}},\;{F1}_{k}=\frac{2\left(+P_{k}\right)\cdot SE_{k}}{\left(+P_{k}\right)+SE_{k}},\\ Acc=\frac{\sum_{k=1}^{m}M_{kk}}{\sum_{k=1}^{m}\sum_{l=1}^{m}M_{kl}},\;Kappa=\frac{Acc-EA}{1-EA}, \end{array}$$

respectively, where EA means the expected accuracy and is defined by

(16) $$\begin{array}{l} EA=\frac{\sum_{p=1}^{m}\left(\sum_{q=1}^{m}M_{pq}\right)\times \left(\sum_{q=1}^{m}M_{qp}\right)}{{\left(\sum_{p,q=1}^{m}M_{pq}\right)}^{2}}. \end{array}$$

When *m* = 3, *k* = 1 means wake, *k* = 2 means REM, and *k* = 3 means NREM. When classifying wake and sleep stages, *k* = 1 means wake, and *k* = 2 means sleep; when classifying REM and NREM stages, *k* = 1 means REM, and *k* = 2 means NREM. When *m* = 2, *SE*_1_ is reduced to the usual sensitivity (SE), *SE*_2_ is reduced to the usual specificity (SP), and +*P*_1_ is reduced to the precision (PR). For each database and each performance measurement, we report the mean ± standard deviation of all subjects.

All experiments in this and next sections were done using Windows 7 operating system environment equipped with i5-4570 CPU and 32 GB RAM. Under this computational environment, given a random seed, the whole training process of an SVM model takes 5–7 min on average. For the reproducibility purpose, the Matlab code is available in the GitHub repository website^3^.

#### 4.4.3. Automatic Sleep Stage Classification Result

We performed three classification tasks—sleep v.s. wake, REM v.s. NREM, and finally wake v.s. REM v.s. NREM. The random seed is fixed to 1 in all cases when we ran the subsampling scheme. The results are shown in Tables 1–3, where the SVM model was trained on the CGMH-training dataset and tested on CGMH-validation, DREAMS, and UCDSADB, respectively. For the interested readers, we also include extensive experimental results with different settings in Supplementary Material (section 3), such as results of training on different datasets, and different random seeds. All results are similar to those reported in the main article.

**Table 1:** SVM cross-database performance of subjects for Wake and Sleep classification with a single random seed.

	**CGMH-training**	**CGMH-validation**	**DREAMS**	**UCDSADB**
TP	76 ± 43	76 ± 44	101 ± 55	85 ± 44
FP	151 ± 49	126 ± 48	175 ± 61	149 ± 59
TN	462 ± 68	449 ± 102	592 ± 112	448 ± 110
FN	27 ± 33	42 ± 43	56 ± 45	73 ± 52
SE (%)	78.3 ± 14.7	70.9 ± 16.0	66.9 ± 16.1	57.6 ± 15.5
SP (%)	76.0 ± 6.1	78.9 ± 5.4	77.6 ± 5.8	75.3 ± 5.5
Acc (%)	75.2 ± 5.4	75.8 ± 4.4	74.7 ± 5.0	70.6 ± 5.4
PR (%)	34.0 ± 17.0	38.1 ± 19.6	37.0 ± 18.8	35.6 ± 17.3
F1	0.438 ± 0.161	0.452 ± 0.140	0.445 ± 0.146	0.407 ± 0.140
AUC	0.839 ± 0.084	0.824 ± 0.090	0.789 ± 0.090	0.702 ± 0.094
Kappa	0.320 ± 0.146	0.322 ± 0.123	0.308 ± 0.148	0.238 ± 0.133

*The training database is CGMH-training. For each database and each performance measurement, we report the mean ± standard deviation of all subjects*.

Table 1 lists the result of classifying wake and sleep stages with different testing sets. For each testing database, we show the mean±standard deviation of each prediction outcome measurement of all subjects in that database. Table 1 shows the performances of training the model on CGMH-training and testing it on CGMH-validation, DREAMS, and UCDSADB. When considering the CGMH database, the (SE, SP) pair for CGMH-training and CGMH-validation are (78.3±14.7%, 76.0±6.1%) and (70.9±16.0%, 78.9±5.4%), respectively. When testing on DREAMS, the (SE, SP) pair becomes (66.9±16.1%, 77.6±5.8%). SP remains in the range of 70%, although SE falls below 70%. This result of the cross database testing is similar to that of validation result. When tested on UCDSADB, the pair of (SE, SP) becomes (57.6±15.5%, 75.3±5.5%). The overall performance on UCDSADB drops as expected since it contains sleep apnea subjects, and their sleep dynamics is disturbed by the sleep apnea. Overall, the cross-database validation results suggest that our model does not overfit. Moreover, we found that the down-sampling scheme alleviates the imbalance database issue.

Table 2 shows the performance for the REM and NREM classification. In this task, since the number of NREM epochs is much more than that of REM epochs, we apply the same down-sampling process to NREM as discussed in section 4.4.2. Tables 1, 2 have several similarities.

**Table 2:** SVM cross-database performance for REM and NREM classification with a single random seed in the training procedure.

	**CGMH-training**	**CGMH-validation**	**DREAMS**	**UCDSADB**
TP	75 ± 31	68 ± 30	93 ± 34	64 ± 34
FP	113 ± 32	106 ± 43	138 ± 51	133 ± 35
TN	400 ± 68	391 ± 94	490 ± 97	373 ± 68
FN	25 ± 23	20 ± 17	46 ± 27	50 ± 37
SE (%)	76.3 ± 16.0	78.1 ± 17.4	67.5 ± 16.7	58.0 ± 18.0
SP (%)	78.0 ± 4.8	79.6 ± 6.5	78.4 ± 5.2	73.7 ± 5.8
Acc (%)	77.4 ± 5.6	77.8 ± 8.3	76.3 ± 6.4	70.4 ± 6.4
PR (%)	39.4 ± 13.6	41.4 ± 19.0	41.0 ± 15.4	31.9 ± 16.0
F1	0.505 ± 0.138	0.510 ± 0.160	0.503 ± 0.144	0.390 ± 0.156
AUC	0.842 ± 0.094	0.849 ± 0.108	0.796 ± 0.115	0.711 ± 0.120
Kappa	0.382 ± 0.150	0.393 ± 0.175	0.312 ± 0.175	0.227 ± 0.162

*The training database is CGMH-training. The subject #24 in CGMH-validation and subject #9 in UCDSADB were dropped because they do not have REM epochs. For each database and each performance measurement, we report the mean ± standard deviation of all subjects*.

Finally, Table 3 shows the performance for the wake, REM, and NREM classification. In this experiment, since the number of NREM is much more than those of wake and REM, the down-sampling scheme is applied to NREM. The Acc's in all cases are about 60%, except the UCDSADB. The SE's of wake, REM, and NREM are balanced and consistent across databases, except UCDSADB. Again, this result might be due to the fact that UCDSADB contains subjects with sleep apnea. On the other hand, note that the +P of NREM is higher than other classes, which is expected due to the dependence of +P on the database prevalence. In Supplementary Material, we provide more cross-database validation results.

**Table 3:** SVM cross-database performance for Wake, REM, and NREM classification with a single random seed in the training procedure.

	**CGMH-training**	**CGMH-validation**	**DREAMS**	**UCDSADB**
SE (%) (Wake)	63.7 ± 15.3	61.1 ± 19.0	56.2 ± 14.5	39.5 ± 11.9
SE (%) (REM)	62.8 ± 17.4	67.1 ± 20.9	57.0 ± 18.0	48.4 ± 19.6
SE (%) (NREM)	71.9 ± 6.7	72.6 ± 6.4	72.5 ± 6.8	66.0 ± 7.6
+P (%) (Wake)	40.6 ± 19.1	43.7 ± 16.6	44.1 ± 18.3	39.3 ± 17.9
+P (%) (REM)	39.1 ± 14.9	40.0 ± 17.7	39.4 ± 15.5	28.5 ± 15.8
+P (%) (NREM)	89.3 ± 8.8	85.6 ± 16.0	83.7 ± 8.6	76.6 ± 9.2
Acc (%)	68.3 ± 6.4	67.6 ± 9.2	66.3 ± 6.4	57.1 ± 7.1
Kappa	0.401 ± 10.2	0.390 ± 0.117	0.372 ± 0.116	0.244 ± 0.108

*The training database is CGMH-training. The subject #24 in CGMH-validation and the subject #9 in UCDSADB were dropped because they do not have REM epochs. For each database and each performance measurement, we report the mean ± standard deviation of all subjects*.

## 5. Discussion and Conclusion

In this work, the TDA tools are considered to analyze time series. Specifically, we propose a set of novel persistence statistics features to quantify HRV by analyzing IHR time series by TDA tools. The proposed HRV features are applied to predict sleep stages, ranging from wake, REM, and NREM. In addition to being computationally efficient, the algorithm is theoretically sound supported by mathematical and statistical results. Note that while we focus on the HRV analysis for the sleep stage annotation, the proposed algorithm has a potential to be applied to analyze other time series and study the HRV for other clinical problems.

### 5.1. Theoretical Supports and Open Problems

We find that empirically, *M* and *L* are simple yet effective representations of the persistence diagram and reveal signatures about the underlying object. It would be interesting to investigate, in theory, the probability distribution of *M* and *L* for a given simplicial complex. However, to the best of our knowledge, while there have been several works in this direction, it is still a relatively open problem. Recently, there has been some theoretical work toward this direction, namely the theory of random complexes (see e.g., survey papers Kahle, 2014; Bobrowski and Kahle, 2018 and references therein). In order to understand the role of noises in the persistence diagram, there have been studies on the topology of the noise. In the theory of random fields, authors in Mischaikow et al. (2010) used sub-level sets as filtration to study the number of components (β_0_) with various random processes; in Adler et al. (2010), authors studied the relation between random fields and the persistent homology in general. In particular, as mentioned in Adler et al. (2010): “It would be interesting to know more about the real distributions lying behind the persistence diagram, but at this point we know very little.” There is also a result in random cubical complexes (Hiraoka and Tsunoda, 2018), and a few work on the limiting theorem of total sum persistence (Owada, 2018) and persistence diagrams (Hiraoka and Tsunoda, 2018). It would also be interesting to study the stability of each persistence statistics. As of now, only the sum of *L*, the max of *L*, and the entropy of *L* have been shown to be stable (Cohen-Steiner et al., 2010; Atienza et al., 2020). However, the rest of persistence statistics is still unknown. Another interesting direction, instead of focusing on each persistence statistics, is to study the probability distributions of *M* and *L*. For instance, let ρ_*M*_ and ρ_*L*_ be the empirical probability density function of the sets *M* and *L*, respectively. For *S* = *M* or *S* = *L*, could one establish $∥\rho_{S}\left(f\right)-\rho_{S}\left(g\right){∥}_{D}\leq d_{B}\left(P_{q}\left(f\right),P_{q}\left(g\right)\right)$, where ||·||_*D*_ is some suitable statistical distance? We leave those interesting theoretical problems to future work.

### 5.2. Comparison With Existing Automatic Sleep Stage Annotation Results

There have been several results in automatic sleep stage annotation by taking *solely* the HRV into account. A common conclusion is that classifying sleep-wake by quantifying HRV is a challenging job. In general, due to the heterogeneity of the data sets, various evaluation criteria and different features and models used in these publications, it is difficult to have a direct comparison. But to be fair, below we summarize some related existing literatures for a discussion. To the best of our knowledge, except (Malik et al., 2018), there is no result reporting a cross-database validation. For those running validation on a single database, we shall distinguish two common cross validation (CV) schemes – *leave-one-subject-out* CV (LOSOCV) and *non-LOSOCV*. When the validation set and the training set come from different subjects, we call it the LOSOCV scheme; otherwise we call it the non-LOSOCV scheme. The LOSOCV scheme is in general challenging due to the uncontrollable inter-individual variability, while the non-LOSOCV scheme tends to over-estimate. Therefore, for a fair comparison, below we only summarize papers considering *only* the IHR features and carrying out the LOSOCV scheme.

In Xiao et al. (2013), the database was composed of healthy participants aged 16 − 61 years. A random forest model was established to differentiate between the wake, REM, and NREM stages for those epochs labeled as “stationary.” Based on the confusion matrix provided in Xiao et al. (2013), the SE, SP, Acc, and F1 for detecting the wake stage are 51.2, 90.2, 84.0, and 0.50%. The authors also provided the SE of wake, REM, and NREM, which are 53.72±20.15, 59.01±19.72, and 79.50±7.82% respectively. In Table 3, our validation on CGMH-validation is 61.1±19.0, 67.1±20.9, and 72.6±6.4%, respectively. Observe that our SE of wake and REM are better, and SE of NREM is on the similar level. In addition to the balance of all classes due to the sub-sampling scheme in our result, note that we focus on all epochs but not “stationary epochs,” and the subjects in CGMH-validation are not healthy but simply without sleep apnea.

In Mendez and Matteucci (2010), the database was composed of 24 participants aged 40 − 50 years with 0 AHI. The authors took the temporal information and the phase and magnitude of the “sleepy pole” as features to train a hidden Markov model to differentiate REM and NREM stages. The reported SE, SP, and Acc were 70.2, 85.1, and 79.3%, respectively. Our results outperform theirs. Our SE, SP and Acc of the REM and NREM classification in CGMH-validation shown in Table 2 are 78.1±17.4, 79.6±6.5, and 77.8±8.3%, respectively. Observe that both Accs are similar which means portion of correct predictions are similar. Not only our SE is better, but SE and SP are also balanced.

In Lewicke et al. (2008), the database is composed of 190 infants. A variety of features and classification algorithms were considered and the wake and sleep classes were balanced for the analysis. The SE and SP of their multi-layer perceptron model without rejection was 79.0 and 77.5%, respectively. In Table 1, the SE and SP of our result on CGMH-validation is 70.9±16.0 and 78.9±5.4%. Our performance is comparable to theirs. However, there is a fundamental difference between their experiments and ours—the sleep dynamics of infants and adults are different.

In Aktaruzzaman et al. (2015), the database is composed of 20 participants aged 49-68 years with varying degrees of sleep apnea. Detrended fluctuation analysis and a feed-forward neural network were applied to differentiate the wake and sleep stages. Various epoch lengths were considered, and the highest performance was recorded on an epoch length of 5 min. The Acc, SE, SP, and Cohen's kappa were 71.9±18.2, 43.7±27.3, 89.0±7.8, and 0.29±0.24, respectively. We consider UCDSADB for a comparison. In Table 1, the Acc, SE, SP, and Cohen's kappa of our testing result on UCDSADB is 70.6±5.4, 57.6±15.5, 75.3±5.5, and 0.238±0.133%. Their Acc and ours are on the same level, our SE is better than theirs, while their SP is better than ours. However, our SE and SP are balanced compared with theirs. A major difference is that our standard deviations for Acc, SE, SP are much smaller. Thus, our performance is comparable to theirs.

In Long et al. (2012), fifteen participants aged 31.0±10.4 years with the Pittsburgh Sleep Quality Index less than 6 were considered. The linear discriminant-based classifier was trained with spectral HRV features. The SE, SP, Cohen's kappa and AUC were 49.7±19.2%, 96±3.3%, 0.48±0.24 and 0.54, respectively. As shown in Table 1, the SE, SP, Cohen's kappa and AUC of our result on CGMH-validation is 70.9±16.0, 78.9±5.4, 0.322±0.123, and 0.824±0.090%, respectively. Again, compared with their results, our SE and SE are more balanced.

To make a conclusion, we emphasize that all those results under comparison are not carried out in the cross-database scheme. Also, usually the SE and SP are not balanced with high SP, which leads to the high accuracy. Therefore, the results suggest that the proposed persistence statistics features and chosen learning model lead a better, or at least similar, performance compared with the state-of-the-art results. The cross-database validation further suggests the usability of the persistence statistics features and the proposed learning scheme in clinical setups. Last but not the least, due to the numerical efficiency of the proposed persistence statistics features, it is potential to apply it to analyze large scale time series.

### 5.3. Technical Issues

We remark that although it is possible to include $P_{i}\left(R_{120,1}\left({\text{x}}^{\left(k,j\right)}\right)\right)$ for *i* ≥ 2 in (14), in practice, it is a challenging task due to its computational complexity. Its computation is known to be poorly scalable in dimension and memory-intensive. We refer readers to Otter et al. (2017) for more details and comparisons among state-of-arts TDA packages and extensive benchmark. To get an idea of the computational cost, for any epoch, the computational time by the state-of-art package Ripser for $P_{1}\left(R_{120,1}\left({\text{x}}^{\left(k,j\right)}\right)\right)$, $P_{2}\left(R_{120,1}\left({\text{x}}^{\left(k,j\right)}\right)\right)$, and $P_{3}\left(R_{120,1}\left({\text{x}}^{\left(k,j\right)}\right)\right)$ are 0.06, 1.7, and 106 s in a standard laptop, respectively. This echos the fact that the computation of $P_{i}\left(R_{120,1}\left({\text{x}}^{\left(k,j\right)}\right)\right)$ does not scale well in *i* Otter et al. (2017). We demonstrated on adding features $P_{2}\left(R_{120,1}\left({\text{x}}^{\left(k,j\right)}\right)\right)$ and tested classification performance on DREAMS and UCDSADB datasets. The results are listed in Supplementary Tables 18, 19. Comparing Supplementary Table 18 with Supplementary Table 1 and Supplementary Table 19 with Supplementary Table 2, we find that the improvements are too marginal to justify the additional computational time.

Thus, it would be inefficient to obtain the higher dimensional persistence features. A possible approach for tackling the computational inefficiency is to reduce the Taken's embedding dimension. In Myers et al. (2019), the authors discussed how to find a proper embedding dimension *p* by considering the false nearest neighbors (Fraser and Swinney, 1986). It would be interesting to combining this embedding technique to our future works. Also, finding another reduction criterion for the Taken's embedding is also an important future direction.

### 5.4. Limitations and Future Directions

In addition to the theoretical development discussed above, there are several interesting practical problems left untouched. While we systematically consider the inter-individual variance, the race, the machine, and the sleep disorder by taking three different databases into account, we acknowledge the fact that the data is collected from the sleep lab. When the data is collected from the real-world mobile device, it is not clear if the algorithm could perform as well and run in real-time. Moreover, its performance for the home-based screening needs to be further evaluated. Yet, in the current mobile health market, the photoplethysmography (PPG) sensor has been widely applied, and its applicability for the sleep-wake classification has been reported in Malik et al. (2018). It is interesting to see how the TDA approach could be applied to analyze the HRV from the PPG for the sleep stage classification mission. From the data analysis perspective, it would be interesting to perform a more sophisticated analysis and take other features from the persistence diagram. For instance, the persistent homology transformation (Turner et al., 2014b) was recently developed and proven to be a sufficient statistic, and had been successfully applied to the shape analysis. It would be interesting to combine the persistent homology transformation and persistence statistics. IHR is a well-known non-stationary time series. Based on the encouraging results of applying the TDA, we suspect that the persistence statistics features could be applied to study other clinical problems related to HRV, and furthermore, analyze other physiological time series, including the multivariate ones. There has been some work using TDA tools to analyzing the multivariate time series (Merelli et al., 2015; Gidea and Katz, 2018; Wu and Hargreaves, 2021) where the critical step is to transform multivariate time series into a point cloud so that Vietoris-Rips complex persistent homology can be computed. It would also be interesting to investigate ways to use the sublevel set filtration in this context. We will explore those limitations/directions in our future work.

## Data Availability Statement

The original contributions presented in the study are included in the article/supplementary material, further inquiries can be directed to the corresponding author/s.

## Author Contributions

Y-MC initiated the project, devised the main pipeline, prototyped the code, and wrote the manuscripts. C-SH implemented the codes, ran all empirical results, produced all the tables, and wrote the manuscripts. Y-LL processed the datasets, interpreted the results, and provided the feedback on physiological aspects. H-TW initiated the project, interpreted the results, wrote the manuscripts, and provided the feedback on all aspects of the project. All authors contributed to the article and approved the submitted version.

## Conflict of Interest

The authors declare that the research was conducted in the absence of any commercial or financial relationships that could be construed as a potential conflict of interest.

## Footnotes

^1^doi: 10.5281/zenodo.2650142

^2^https://archive.physionet.org/pn3/ucddb/

^3^https://github.com/peterbillhu/TDA_for_SleepWake_Classifications

## Abbreviations

AASM: American academy of sleep medicine
Acc: accuracy
AHI: apnea-hypopnea index
ANS: autonomic nervous system
AUC: area under curve
CGMH: Chang Gung Memorial Hospital
CV: cross validation
DREAMS: DREAMS subjects database
ECG: electrocardiogram
ECOC: error-correcting output codes
EEG: electroencephalogram
EOG: electrooculogram
EMG: eletromyogram
HRV: heart rate variability
IHR: instantaneous heart rate
LOSOCV: leave-one-subject-out CV
NREM: non-rapid eyeball movement
PPG: photoplethysmography
PR: precision
PS: persistence statistics
PSG: polysomnogram
REM: rapid eyeball movement
RRI: R peak to R peak interval
SE: sensitivity
SP: specificity
SVM: support vector machine
TDA: topological data analysis
UCDSADB: St. Vincent's University Hospital/University College Dublin Sleep Apnea Database
VR: vietoris-rips.
