# Supplementary material for: A Persistent Homology Approach to Heart Rate Variability Analysis With an Application to Sleep-Wake Classification
Source: Front Physiol. 2021 Mar 1;12:637684. doi: 10.3389/fphys.2021.637684 (PMC7959762; doi:10.3389/fphys.2021.637684)
Supplement: Supplementary file 1 [file Presentation_1.pdf]

frontiers

Supplementary Material for "A persistent homology approach to heart rate variability analysis with an application to sleep-Wake classification"

SI.1 MORE MATHEMATICAL BACKGROUND

SI.1.1 More Simplicial Complexes

To investigate the complicated structure of an object, an intuitive way is to use simple objects as building blocks to approximate the original object. For instance, in computer graphics, curves and surfaces in Euclidean spaces are approximated by line segments and triangles, *e.g.* Figure SI.2 (D). In TDA, the main building blocks are *simplicial complexes*. Simplicial complexes are important tools to approximate continuous objects via combinatorial objects, such as vertices, edges, faces, and so on. Although simplicial complexes and simplicial homology can be studied in an abstract and general way (see e.g. Edelsbrunner and Harer (2010); Munkres (1993)), to enhance the readability, we present the notion in a relatively concrete way without losing critical information.

We start with introducing simplex. Intuitively, a simplex is a "triangle" in different dimension. As shown in Figure SI.1, vertices, line segments, triangles and tetrahedron in $\mathbb{R}^d$ are $0, 1, 2, 3$-dimensional triangles respectively, and they are called 0-simplexes, 1-simplexes, 2-simplexes and 3-simplexes in $\mathbb{R}^d$ respectively. The formal definitions can be found below.

DEFINITION 1 (Munkres (1993) Sec. 1.1, p. 3). *Let $x_0, x_1, \ldots, x_q$ be affinely independent points in $\mathbb{R}^d$. The q-**simplex**, denoted by $\sigma := \langle x_0, x_1, \ldots, x_q\rangle$, is defined to be the convex hull of $x_0, x_1, \ldots, x_q$. In other words,*

$$\sigma = \left\{ \lambda_0 x_0 + \lambda_1 x_1 + \cdots + \lambda_q x_q \;\middle|\; \lambda_0, \lambda_1, \ldots, \lambda_q \in [0,1], \sum_{i=0}^{q} \lambda_i = 1 \right\}.$$

We denote $\mathrm{Vert}(\sigma) := \{x_0, x_1, \ldots, x_q\}$.

Recall that $x_0, x_1, \ldots, x_q$ are affinely independent points in $\mathbb{R}^d$ if and only if $x_1 - x_0, \ldots, x_q - x_0$ are linearly independent vectors in $\mathbb{R}^d$. Any q-simplex is a q dimensional object, and it consists of lower degree simplexes. We are interested in the relation among simplexes of different dimensions. Since any subset V of $\mathrm{Vert}(\sigma)$ is also affinely independent, the convex hull of V also forms a simplex with dimensional $|V| \leq q$ where $|V|$ denotes the cardinality of V. This lower dimensional simplex is called a *face* of σ.

DEFINITION 2 (Munkres (1993) Sec. 1.1, p. 5). *Let σ be a q-simplex, and $V \subset \mathrm{Vert}(\sigma)$. The convex hull of V is called a* face *of σ. Moreover, if $|V| = k$, then the face $\tau = \langle V\rangle$ is called a k-face of σ.*

For example, in Figure SI.1, the 3-simplex (tetrahedron) $\langle G, H, I, J\rangle$ has the following faces:

- 0-face: $\langle G\rangle$, $\langle H\rangle$, $\langle I\rangle$ and $\langle J\rangle$;
- 1-face: $\langle G, H\rangle$, $\langle G, I\rangle$, $\langle G, J\rangle$, $\langle H, I\rangle$, $\langle H, J\rangle$ and $\langle I, J\rangle$;
- 2-face: $\langle G, H, I\rangle$, $\langle G, I, J\rangle$, $\langle G, H, J\rangle$ and $\langle H, I, J\rangle$.

A *simplicial complex* $\mathcal{K}$ in $\mathbb{R}^d$ is a collection of finite simplexes σ in $\mathbb{R}^d$ so that any intersection of two arbitrary simplexes is a face to each of them. It is formalized in the following definition.

DEFINITION 3 (Munkres (1993) Sec. 1.2, p. 7). *A collection $\mathcal{K}$ of simplexes in $\mathbb{R}^d$ is said to be a simplicial complex if it satisfies the following two properties:*

- *If $\sigma \in \mathcal{K}$ and τ is a face of σ, then $\tau \in \mathcal{K}$;*
- *If $\sigma_1, \sigma_2 \in \mathcal{K}$, then $\sigma_1 \cap \sigma_2$ is a face of σ_1 and σ_2. In particular, $\sigma_1 \cap \sigma_2 \in \mathcal{K}$.*

For instance, the surface in Figure SI.2(D) is represented by a simplicial complex consisting of simplexes with maximal dimension 2, that is, triangles and their edges and vertices.

In order to study the topological information of a given simplicial complex, we study relations among different dimensional simplexes. It can be understood as a way to measure the complexity of simplicial complexes. Homology theory is the main theory we count on to achieve this goal. In the end, we will define the Betti numbers.

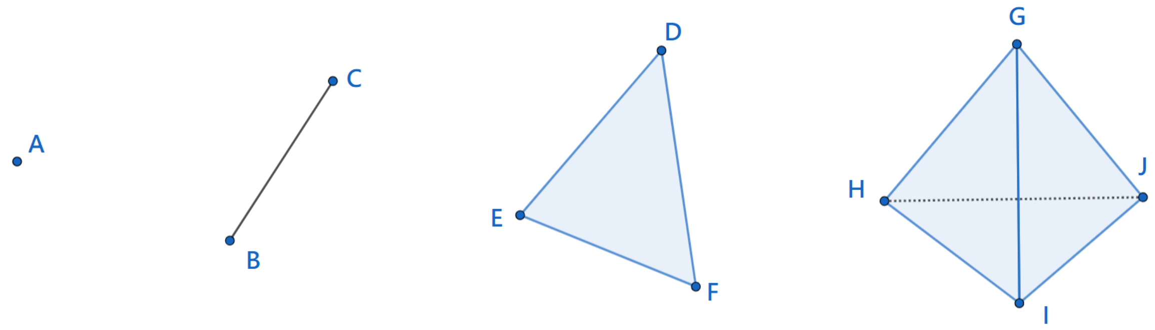

Figure SI.1: Illustration of simplices in dimension $0, 1, 2, 3$. The singleton point $\{A\}$ forms a 0-simplex and convex hulls $\langle B, C\rangle$, $\langle D, E, F\rangle$ and $\langle G, H, I, J\rangle$ are 1-simplex, 2-simplex, 3-simplex in an Euclidean space respectively.

SI.1.2 More on Betti numbers and Homology

Homology is a classic subject in algebraic topology Munkres (1993), which captures "holes" of geometric objects in different dimensions. While we can discuss homology in more general geometric objects, in this work, we mainly consider simplicial complexes as our target object. We now discuss how to quantify q-dimensional holes.

In order to count q-dimensional holes in $\mathcal{K}$, we need to find the interactions among different simplexes. To achieve it, one adds an algebraic structure to simplexes. Formally, given q-simplex $\sigma_1, \sigma_2, \ldots, \sigma_n \in \mathcal{K}$, one could write a formal sum as $c = \sum_{i=1}^{n} \nu_i \sigma_i$, where $\nu_i \in \mathbb{Z}_2$. This formal sum is commonly known as a *q-chain*. One could also define an addition as $\sum_{i=1}^{n} \nu_i \sigma_i + \sum_{i=1}^{n} \mu_i \sigma_i := \sum_{i=1}^{n} (\nu_i + \mu_i)\sigma_i$. We consider the collection of all q-chains, denoted by

$$C_q(\mathcal{K}) := \left\{ \sum_{i=1}^{n} \nu_i \sigma_i \,\middle|\, \nu_i \in \mathbb{Z}_2,\ \sigma_i \in \mathcal{K},\ \dim(\sigma) = q \right\}. \tag{SI.1}$$

One could prove that $C_q(\mathcal{K})$ is actually a vector space over $\mathbb{Z}_2$ with the above addition. For example, consider the simplicial complex $\mathcal{K} = \langle D, E, F\rangle$ in Figure SI.1. $\langle D\rangle + \langle E\rangle$ is an element in $C_0(\mathcal{K})$, and $\langle D, E\rangle + \langle E, F\rangle$ is an element in $C_1(\mathcal{K})$. Note that $\langle D, E\rangle + \langle E\rangle$ is not defined because they live in different spaces. There is a natural relation between $C_q(\mathcal{K})$ and $C_{q-1}(\mathcal{K})$, called the *boundary map*.

DEFINITION 4 (Munkres (1993) Sec. 1.5, p. 30). *Let* $\sigma = \langle x_0, x_1, \cdots, x_q\rangle \in C_q(\mathcal{K})$. *The* q^{th} boundary map $\partial_q : C_q(\mathcal{K}) \to C_{q-1}(\mathcal{K})$ *over* $\mathbb{Z}_2$ *is defined by*

$$\partial_q(\langle x_0, x_1, \cdots, x_q\rangle) = \sum_{i=0}^{q} \langle x_0, \cdots, \widehat{x_i}, \cdots x_q\rangle, \tag{SI.2}$$

where $\sigma = \langle x_0, x_1, \cdots, x_q\rangle$ *being a q-simplex in* $\mathcal{K}$ *and the* $\widehat{\bullet}$ *denotes the drop-out operation.*

For instance, $\partial_2(\langle D, E, F\rangle) = \langle E, F\rangle + \langle D, F\rangle + \langle D, E\rangle$. ∂_q captures the boundary of a given simplex, which justifies the nomination. With the boundary maps, there is a nested relation among chains

$$\cdots \xrightarrow{\partial_{n+1}} C_n(\mathcal{K}) \xrightarrow{\partial_n} C_{n-1}(\mathcal{K}) \xrightarrow{\partial_{n-1}} \cdots C_1(\mathcal{K}) \xrightarrow{\partial_1} C_0(\mathcal{K}). \tag{SI.3}$$

This nested relation among chains is known as the *chain complex*, which is denoted as $\mathcal{C} = \{C_q, \partial_q\}_{q\in\mathbb{Z}}$.

A fundamental result in homology theory (Munkres (1993) Lemma 5.3 Sec. 1.5, p. 30) is that the composition of any two consecutive boundary maps is a trivial map, i.e. $\partial_{q-1} \circ \partial_q = 0$. This result allows one to define the following quotient space. We first denote *cycles* and *boundaries* by $Z_q(\mathcal{K})$ and $B_q(\mathcal{K})$, respectively, which are defined as

$$Z_q(\mathcal{K}) := \ker(\partial_q) = \{c \in C_q \mid \partial_q(c) = 0\},$$
$$B_q(\mathcal{K}) := \text{im}(\partial_{q+1}) = \{\partial_{q+1}(z) \in C_q \mid z \in C_{q+1}\}.$$

Note that each $B_q(\mathcal{K})$ is a subspace of $Z_q(\mathcal{K})$. Therefore, we can define the qth *homology* to be the quotient space

$$H_q(\mathcal{K}) := \frac{Z_q(\mathcal{K})}{B_q(\mathcal{K})} = \frac{\ker(\partial_q)}{\text{im}(\partial_{q+1})}, \tag{SI.4}$$

which is again a vector space. Finally, the Betti number is defined to be the dimension of the homology. More precisely,

$$\beta_q(\mathcal{K}) = \dim(H_q(\mathcal{K})). \tag{SI.5}$$

As a result, given a simplicial complex $\mathcal{K}$, the *homology of* $\mathcal{K}$ is a collection of vector spaces $\{H_q(\mathcal{K})\}_{q=0}^{\infty}$, and its *Betti numbers* is denoted as $\beta(\mathcal{K}) := \{\beta_q(\mathcal{K})\}_{q=0}^{\infty}$. Formally speaking, β_q measures the number of q-dimensional holes. For instance, β_0 counts the number of components, β_1 counts the number of loops, and β_2 counts the number of voids. Using this intuition and by visual inspection, one may count the Betti numbers of those simplicial complexes appeared in Figure SI.2.

Figure SI.2 shows some examples of simplicial complexes in $\mathbb{R}^2$, X_1 to X_3, and $\mathbb{R}^3$, X_4 to X_6. For instance, the direct computation shows that $H_0(X_1) = \mathbb{Z}_2$, $H_1(X_1) = \mathbb{Z}_2$, and $H_q(X_1) = \{0\}$ for all $q > 1$; hence $\beta(X_1) = \{1, 1, 0, \ldots\}$. The only nontrivial homology group of X_2 is $H_0(X_2) = \mathbb{Z}_2^2$, and hence $\beta(X_2) = \{2, 0, \ldots\}$. For objects X_4 to X_6, voids in $\mathbb{R}^3$ surrounded by spheres or tori are 2-dimensional

holes, so $H_2(X_5) = \mathbb{Z}_2^5$ and $H_2(X_6) = \mathbb{Z}_2$. Note that $H_1(X_6) = \mathbb{Z}_2^2$ because a torus has two 1-dimensional holes (Munkres (1993) Theorem 6.2 Sec. 1.6, p. 35).

SI.1.3 More about sub-level set and VR filtrations

First, we provide an example of the sublevel set filtration.

EXAMPLE SI.1.3.1. *Consider a simple filtration $\{f_{1.5}, f_{2.5}, f_3\}$ as shown in Figure SI.3. When $h = 1.5$, the sub-level set $f_{1.5}$ has $\beta_0 = 2$ since it contains two connected components i.e., disjoint intervals in $\mathbb{R}^1$. When $h = 2.5$, two connected components in $f_{1.5}$ merged to $f_{2.5}$. Moreover, there is a new interval ($\approx [0.8, 0.9]$) appeared in $f_{2.5}$, and hence $\beta_0(f_{2.5}) = 2$. Finally, when h is lifted to 3, previous intervals are merged to f_3 and we get $\beta_0(f_3) = 1$. This filtering process can be depicted in the PD as shown in Figure SI.3(D), which is $\mathcal{P}_0(\{f_{1.5}, f_{2.5}, f_3\}) = \{(1.5, \infty), (1.5, 2.5), (2.5, 3)\}$.*

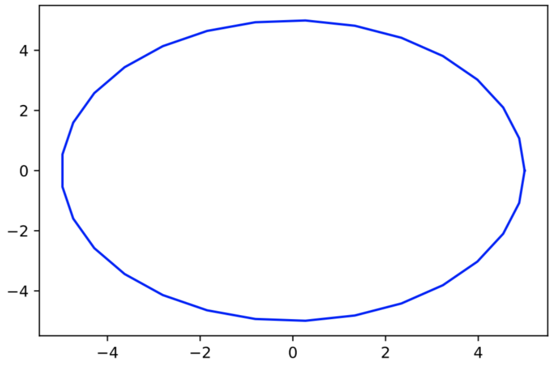

(a) X_1 = one circle

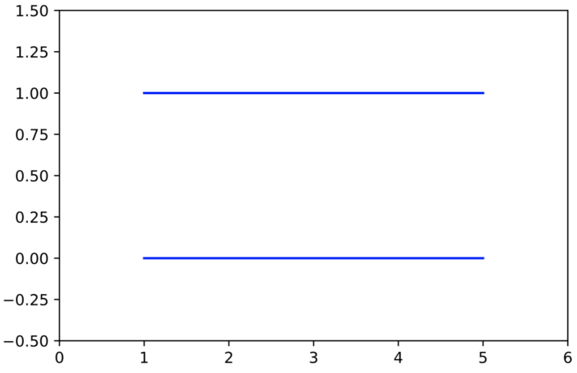

(b) X_2 = two segments

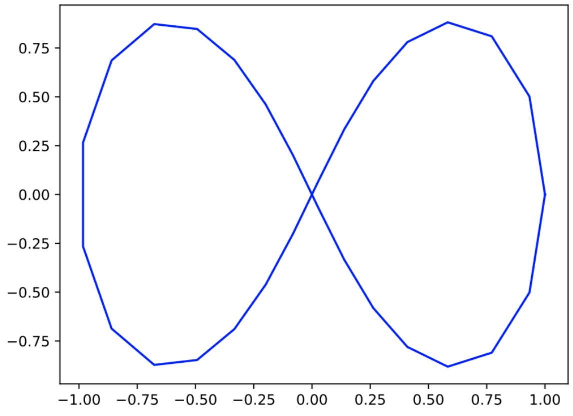

(c) X_3 = curve of "∞"

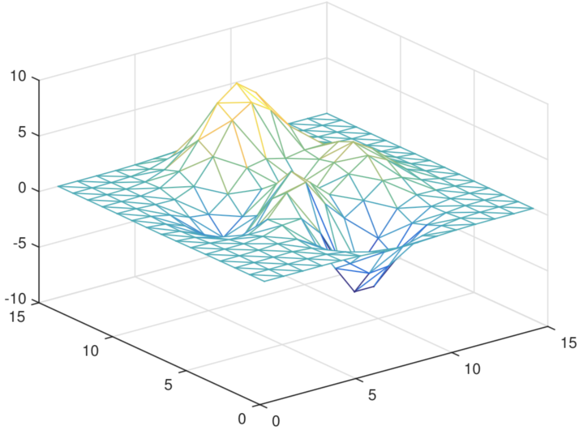

(d) X_4 = a surface

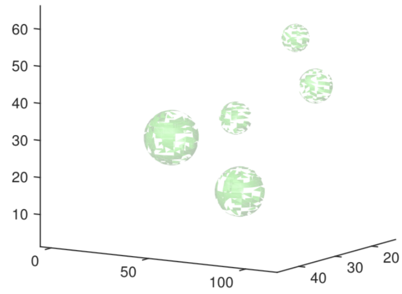

(e) X_5 = five shperes

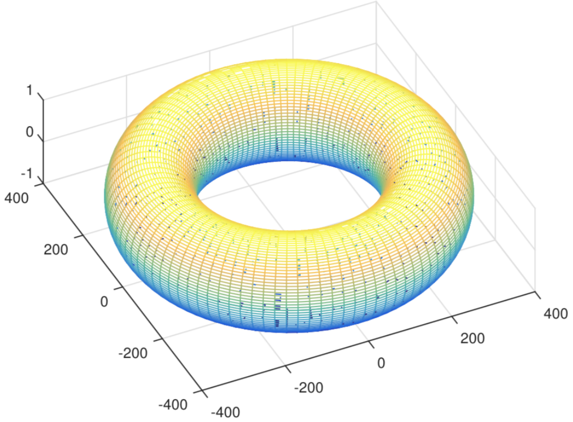

(f) X_6 = torus

Figure SI.2: Some examples of geometric objects in $\mathbb{R}^2$ and $\mathbb{R}^3$. The Betti numbers are: $\beta(X_1) = (1, 1)$, $\beta(X_2) = (2, 0)$, $\beta(X_3) = (1, 2)$, $\beta(X_4) = (1, 0, 0)$, $\beta(X_5) = (5, 0, 5)$ and $\beta(X_6) = (1, 2, 1)$.

Next, we show an illustrative example of the VR filtration.

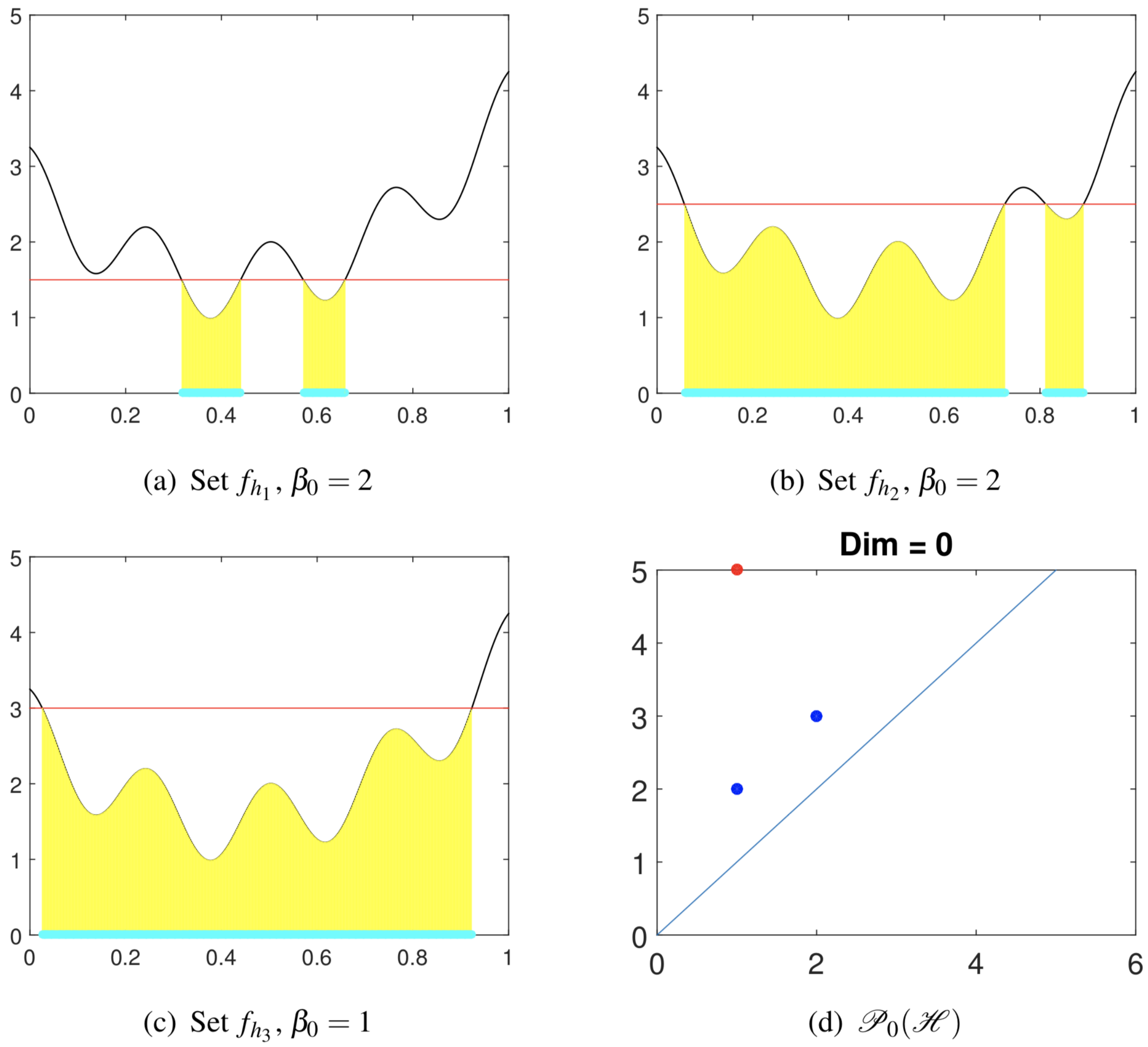

(a) Set f_{h_1}, $\beta_0 = 2$

(b) Set f_{h_2}, $\beta_0 = 2$

(c) Set f_{h_3}, $\beta_0 = 1$

(d) $\mathscr{P}_0(\mathscr{H})$

Figure SI.3: An example of sub-level sets of signal $f(t) = 1 + t + 7(t - 0.5)^2 + \cos(8\pi t)/2$. Unions of blue line segments lying on x-axis in terms (a), (b) and (c) are sub-level sets f_{h_1}, f_{h_2} and f_{h_3} ($h_1 = 1.5$, $h_2 = 2.5$, $h_3 = 3$) with corresponding thresholds. We also use the convention $f_{h_1} = I_{1,\text{left}} \cup I_{1,\text{right}}$ and $f_{h_2} = I_{2,\text{left}} \cup I_{2,\text{right}}$ to decompose f_{h_1} and f_{h_2} by disjoint intervals as blue line segments in (a) and (b). Labels of x, y axes in (a), (b) and (c) are arbitrary units.

EXAMPLE SI.1.3.2. *Figure SI.4 shows an example of VR complex of point-cloud in* $\mathbb{R}^2$*, where points are sampled from curve* X_3 *in Figure SI.2. As in Figure SI.4, when* ϵ *is too small, then the information of Betti numbers of the simplicial complex* $\mathrm{VR}(X;\epsilon)$ *is nothing different to original point-cloud. On the other hand, for extremely large* ϵ*, the only non-zero Betti number of* $\mathrm{VR}(X;\epsilon)$ *is* $\beta_0 = 1$*. For example, the only two* 1*-dimension holes in filtration* $\mathcal{K}_1 \subseteq \mathcal{K}_2 \subseteq \cdots \subseteq \mathcal{K}_6$ *of Figure SI.4 were born at time* 5 *(*$\mathcal{K}_5$*) and died at time* 6 *(*$\mathcal{K}_6$*), hence both of them have coordinate* $(5, 6)$*. Moreover, if a q-dimensional hole is still alive in the final object of the filtration, we will use* ∞ *to denote its death value (which indicates the feature never dies). We also use Figure SI.4 to explain the PD. First, in every* $\mathcal{K}_i$*, we encode each component to the*

smallest index of points belonging to the components. For example, because vertices 5 *and* 14 *represent the same connected component* $\langle 5, 14\rangle$ *in* $\mathcal{K}_1$*, we use* 0*-simplex* $\langle 5\rangle$ *to denote the component.*

With the VR complex, the lifespan of each hole can be computed. For example, both of components $\langle 5\rangle$ *and* $\langle 11\rangle$ *in* $\mathcal{K}_1$ *has life span* $(1, 2)$ *because they would be merged into the connected component represented by* $\langle 4\rangle$ *in* $\mathcal{K}_2$*. On the other hand, because the connected component* $\langle 0\rangle$ *is alive in whole filtration, it has lifespan* $(1, \infty)$*. As a result, the PD of filtration in Figure SI.4 can be expressed as*

$$\begin{aligned}\mathcal{P}_0(\mathcal{K}) =\{&(1,\infty)_{\langle 0\rangle}, (1,4)_{\langle 1\rangle}, (1,2)_{\langle 2\rangle}, (1,2)_{\langle 3\rangle}, (1,3)_{\langle 4\rangle}, (1,2)_{\langle 5\rangle},\\ &(1,2)_{\langle 6\rangle}, (1,2)_{\langle 7\rangle}, (1,2)_{\langle 8\rangle}, (1,3)_{\langle 9\rangle}, (1,3)_{\langle 10\rangle}, (1,2)_{\langle 11\rangle},\\ &(1,2)_{\langle 12\rangle}, (1,2)_{\langle 13\rangle}, (1,2)_{\langle 15\rangle}, (1,3)_{\langle 16\rangle}, (1,2)_{\langle 17\rangle}, (1,2)_{\langle 18\rangle}\}\end{aligned} \tag{SI.6}$$

and $\mathcal{P}_1(\mathcal{K}) = \{(5, 6), (5, 6)\}$*. The notations* $\langle *\rangle$ *of lifespans in* (SI.6) *are used for clarifying which component a lifespan belong to.*

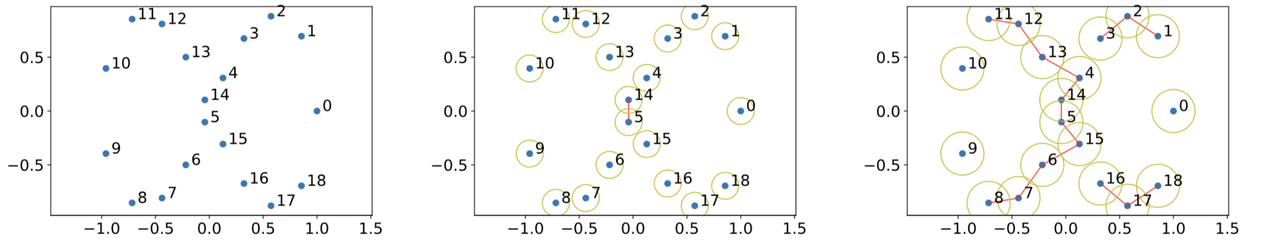

(a) $\mathscr{K}_0 = \mathrm{VR}(X;0)$, $\beta_0 = 19$, $\beta_1 = 0$ (b) $\mathscr{K}_1 = \mathrm{VR}(X;0.25)$, $\beta_0 = 18$, $\beta_1 = 0$ (c) $\mathscr{K}_2 = \mathrm{VR}(X;0.4)$, $\beta_0 = 6$, $\beta_1 = 0$

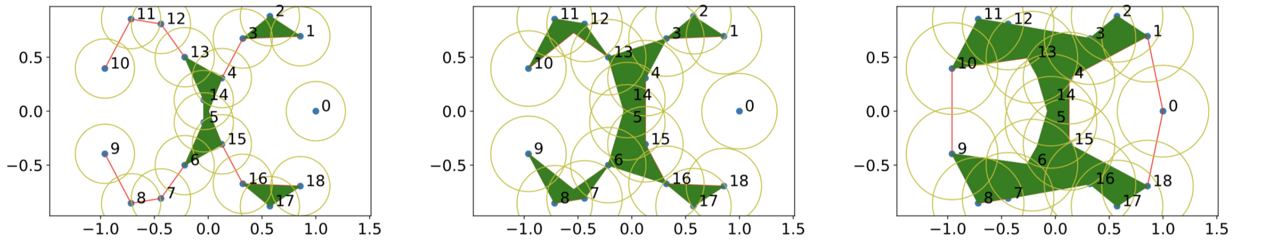

(d) $\mathscr{K}_3 = \mathrm{VR}(X;0.55)$, $\beta_0 = 2$, $\beta_1 = 0$ (e) $\mathscr{K}_4 = \mathrm{VR}(X;0.7)$, $\beta_0 = 2$, $\beta_1 = 0$ (f) $\mathscr{K}_5 = \mathrm{VR}(X;0.85)$, $\beta_0 = 1$, $\beta_1 = 2$

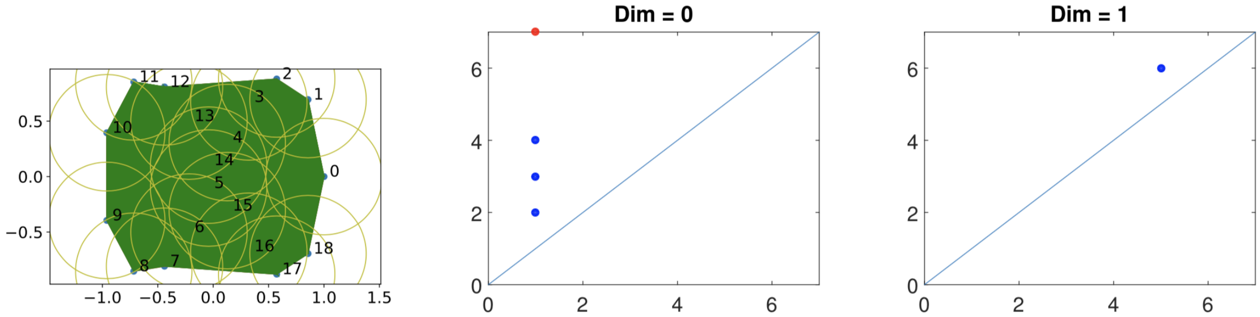

(g) $\mathscr{K}_6 = \mathrm{VR}(X;1.05)$, $\beta_0 = 1$, $\beta_1 = 0$ (h) $\mathscr{P}_0(\mathscr{K})$ (i) $\mathscr{P}_1(\mathscr{K})$

Figure SI.4: An example of filtration made by Vietoris-Rips complex. Labels of x, y axes in $(a) \sim (c)$ are arbitrary units.

SI.2 TAKENS' LAG MAP

In this section, we provide a review of "recovering the manifold" underlying the one dimensional time series. Precisely, we discuss a set of theorems provided in Takens (1981) and an associated embedding algorithm in the time series framework. The algorithm is well-known as the *Takens' lag map* or *lag map*, and has been extensively applied to many fields. In a nutshell, the recovered manifold is the phase space on which the dynamics lives that generates the time series. From now on, denote M to be a d-dim compact manifold without boundary. For the sake of self-containedness, we recall the following definitions.

DEFINITION 5 (Discrete time dynamics). *By a discrete time dynamics, we mean a diffeomorhism $\varphi: M \to M$ with the time evolution $i \mapsto \varphi^i(x_0)$, $i \in \mathbb{N}$, where x_0 is the starting status.*

DEFINITION 6 (Continuous time dynamics). *By a continuous time dynamics, we mean a smooth vector field $X \in \Gamma(M)$ with the time evolution $t \mapsto \gamma_t(x_0)$, where γ_t is the integral curve with respect to X via x_0.*

To simplify the discussion, in both cases, we denote $\Phi_t(x_0)$ to be the time evolution with time $t \in \mathbb{N}$ or $\mathbb{R}$ with the starting point x_0.

DEFINITION 7 (Observed time series). *Let $\Phi_t(x_0)$ be a dynamics on M. The observation is modeled as a function $f : M \to \mathbb{R}$ and the observed time series is $f(\Phi_t(x_0))$.*

The question we have interest is that if we have an observed time series $f(\Phi_t(x_0))$, can we recover M and/or the dynamics? The positive answer and the precise statements are provided in the following two theorems. The proof of these theorems can be found in Takens (1981).

THEOREM SI.2.0.1 (discrete time dynamics). *For a pair (φ, f), $\varphi : M^d \to M^d$ is the C^2-diffeomorphism and $f \in C^2$, it is generic that the map $\Psi : M \to \mathbb{R}^{2d+1}$ given by*

$$\Psi : x \mapsto (f(x),\, f(\varphi(x)),\, f(\varphi^2(x)) \ldots f(\varphi^{2d}(x)))^T \in \mathbb{R}^{2d+1}$$

is an embedding.

By *generic*, we mean an open dense subset of all possible pairs (φ, f). We mention that the theorems hold for non-compact manifolds if f is proper. These technical assumptions are practically assumed held for real world datasets.

THEOREM SI.2.0.2 (Continuous time dynamics). *When $X \in C^2(\Gamma M)$ and $f \in C^2(M)$, it is generic that $\Psi : M \to \mathbb{R}^{2d+1}$ given by*

$$\Psi : x \mapsto (f(x),\, f(\gamma_1(x)),\, f(\gamma_2(x)) \ldots f(\gamma_{2d}(x)))^T \in \mathbb{R}^{2d+1}$$

is an embedding, where $\gamma_t(x)$ is the flow of X of time t via x.

These theorems tell us that we could embed the manifold into a $(2d+1)$ dimensional Euclidean space if we have access to all dynamical behaviors from all points on the manifold. However, in practice the above model and theorem cannot be applied directly. Indeed, for a given dynamical system, most of time we may only have one or few experiments that are sampled at discrete times; that is, we only have access to one or few $x \in M$. We thus ask the following question. Suppose we have the time series

$$\{f(\Phi_{\ell\alpha}(x))\}_{\ell=0}^{N}$$

as our dataset, where $x \in M$ is fixed and inaccessible to us, $\alpha > 0$ is the sampling period, and $N \gg 1$ is the number of samples, what can we do? We first give the following definition.

DEFINITION 8. *The positive limit set (PLS) of x of a vector field $X \in C^2(\Gamma M)$ is defined as*

$$L_c^+(x) := \left\{x' \in M \,|\, \exists\, t_i \to \infty,\ t_i \in \mathbb{R} \text{ such that } \gamma_{t_i}(x) \to x'\right\}$$

and the PLS of x of a diffeomorphism $\varphi : M \to M$ is defined as

$$L_d^+(x) := \left\{x' \in M \,|\, \exists\, n_i \in \mathbb{N} \to \infty, \text{ such that } \varphi^{n_i}(x) \to x'\right\}.$$

It turns out that in this case, we should know whether under generic assumptions the topology and dynamics in the PLS of x is determined by $\{f(\Phi_{\ell\alpha}(x))\}_{\ell=0}^{\infty}$. Precisely, we have the following theorem:

THEOREM SI.2.0.3 (Continuous dynamics with 1 trajectory). *Fix $x \in M$. When $X \in C^2(\Gamma M)$ with flow γ_t passing x, then there exists a residual subset $C_{X,x} \subset \mathbb{R}^+$ such that for all $\alpha \in C_{X,x}$ and diffeomorphism $\varphi := \gamma_\alpha$, the PLS $L_c^+(x)$ for flow γ_t and $L_d^+(x)$ for φ are the same; that is, for all $\alpha \in C_{X,x}$ and for all $q \in L_c^+(p)$, there exists $n_i \in \mathbb{N} \to \infty$ such that $\varphi^{n_i}(x) \to q$.*

This theorem leads to the following corollary, which is what we need to analyze the time series.

COROLLARY SI.2.0.4. *Take $x \in M$, a generic pair $X \in C^2(\Gamma M)$ and $f \in C^2(M)$, and $a \in \mathbb{R}^+$ satisfying generic conditions depending on X and x. Denote the set*

$$\mathcal{P} := \big\{f(\gamma_{k\alpha}(x)),\, f(\gamma_{k\alpha}(x)), \ldots, f(\gamma_{(k+2d)\alpha}(x))\big\}_{k=0}^{\infty}.$$

Then there exists a smooth embedding of M into $\mathbb{R}^{2d+1}$ mapping PLS L_c^+ bijectively to the set $\mathcal{P}$.

Last but not the least, we remark that the noise analysis of the Takens' lag map has been extensively studied, for example Stark et al. (1997).

SI.3 MORE AUTOMATIC ANNOTATION RESULTS

To further illustrate the robustness of our model and features, we perform more cross-database validation.

SI.3.1 Visualize features $\Phi^{(\mathtt{PS})}(\mathcal{P}_i(R_{120,1}))$

The visualization of $\Phi^{(\mathtt{PS})}(\mathcal{P}_i(R_{120,1}))$, where $i = 0, 1$, is shown in Figure SI.5. In order to compare them on the same scale, we perform the standard z-score normalization for each parameter in $\Phi^{(\mathtt{PS})}(\mathcal{P}_i(R_{120,1}))$. We abuse the notation and use the same notation $\Phi^{(\mathtt{PS})}(\mathcal{P}_i(R_{120,1}))$ to denote the normalized parameters. The boxplot of each normalized persistence statistics parameter, where blue (red) bars represent the persistence statistics associated with an IHR time series associated with the sleep (Wake) stage, is shown in Figure SI.5. We performed a rank sum test with a significance level of 0.05 with the Bonferroni correction. We found that there are significant differences between waking and sleeping features for all persistence statistics parameters, except two parameters in $\Phi^{(\mathtt{PS})}(\mathcal{P}_0(R_{120,1}))$. The first principal components of $\bigcup_k\{\Phi^{(\mathtt{PS})}(\mathcal{P}_i(R_{120,1}))\}_{j=1}^{n_k}$ are shown in Figure SI.5(c) and (d). We can observe a separation between sleep and Wake features.

SI.3.2 For the classification of sleep and Wake

We consider the models trained on DREAMS and UCDSADB as shown in Table S1 and Table S2, respectively. In Table S1, we observe that the (SE, SP) pairs are consistent and all are located in the level of $(60, 70)$ except for the UCDSADB. In Table S2, we also observe that the (SE, SP) pairs are all on the level of $(60, 70)$. These consistent performances suggest that our proposed topological features, persistence statistics, are stable and well capture intrinsic characteristics about the sleep and Wake IHR signals.

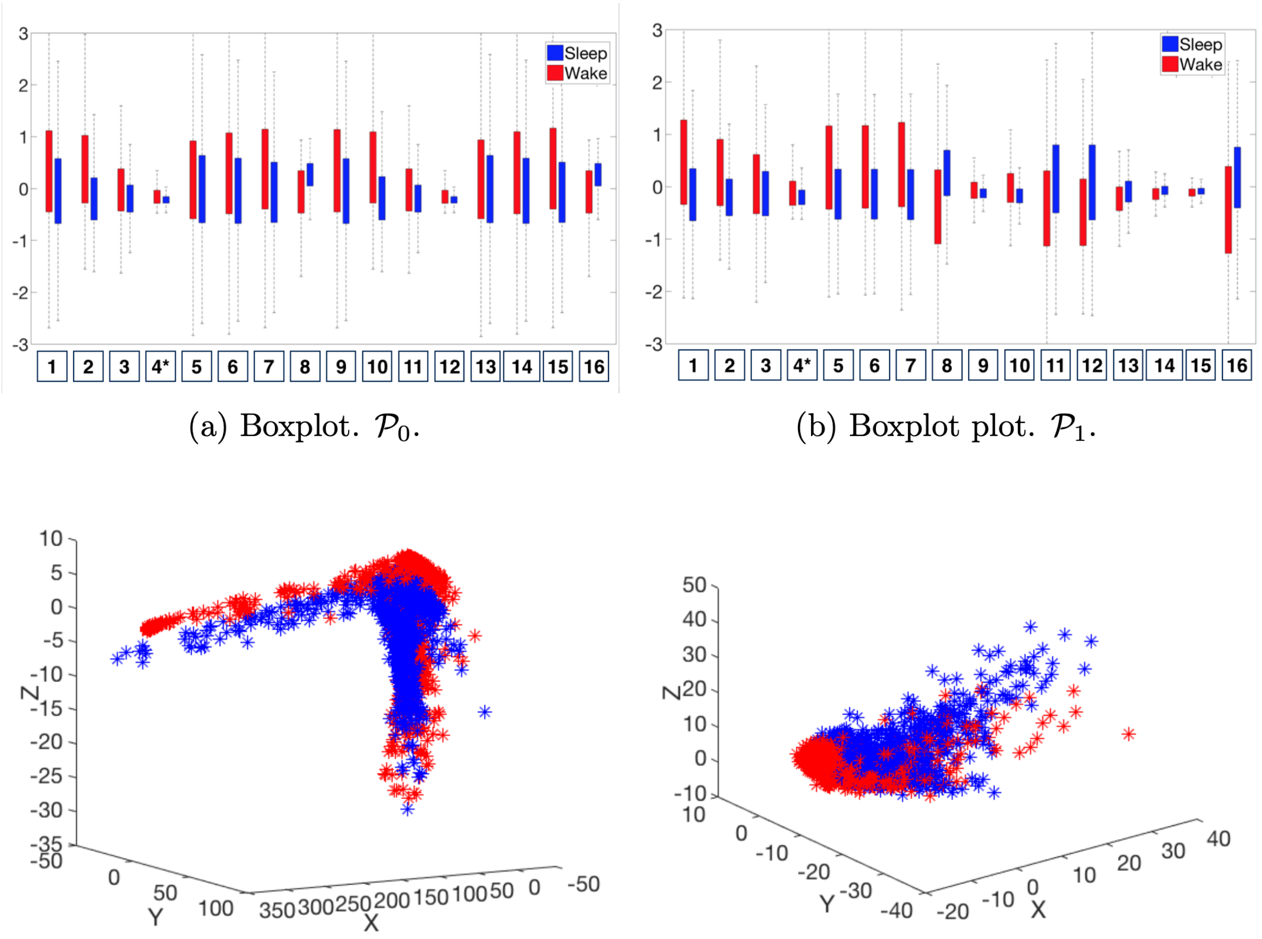

(a) Boxplot. $\mathcal{P}_0$.

(b) Boxplot plot. $\mathcal{P}_1$.

(c) Scatter plot. $\mathcal{P}_0$.

(d) Scatter plot. $\mathcal{P}_1$.

Figure SI.5: Distribution of normalized persistence statistics features, $\Phi^{(\mathrm{PS})}(\mathcal{P}(R_{120,1}))$. (a)-(b) Boxplot of the $\Phi^{(\mathrm{PS})}(\mathcal{P}_i(R_{120,1}))$ where $i = 0, 1$, respectively. The numbers listed on the horizontal axis indicates the number of persistence statistics. * indicates that the feature *fails* to reject the null hypothesis of the significance level of 0.05 on the rank sum test with Bonferroni correction. (c)-(d) Visualization of $\bigcup_k \{\Phi^{(\mathrm{PS})}(\mathcal{P}_i(R_{120,1}))\}_{j=1}^{n_k}$ by the first three principal components.

SI.3.3 For the classification of REM and NREM

We consider the models trained on DREAMS and UCDSADB for the REM and NREM classification. The results are summarized in Tables S5 and S6 respectively. We observe that the overall performance is consistent. As expected, the performance of the model trained on DREAMS is degraded on UCDSADB, and the performance of the model trained on UCDSADB on other databases is slightly lower, particularly the SE's.

Table S1. SVM cross-database performance of subjects for Wake and Sleep classification with a single random seed. The training database is DREAMS. For each database and each performance measurement, we report the mean ± standard deviation of all subjects.

	CGMH-training	CGMH-validation	DREAMS	UCDSADB
TP	74 ± 43	72 ± 40	112 ± 57	86 ± 47
FP	166 ± 55	141 ± 54	176 ± 64	165 ± 63
TN	447 ± 69	434 ± 97	592 ± 112	431 ± 114
FN	29 ± 34	43 ± 44	45 ± 41	71 ± 54
SE (%)	75.9 ± 15.7	70.7 ± 17.0	73.7 ± 15.0	58.6 ± 16.9
SP (%)	73.5 ± 7.1	76.5 ± 6.3	77.6 ± 6.0	72.5 ± 7.1
Acc (%)	72.7 ± 6.7	73.6 ± 4.7	75.9 ± 5.3	68.7 ± 7.7
PR (%)	31.6 ± 18.3	35.9 ± 18.9	39.2 ± 18.1	33.6 ± 17.3
F1	0.411 ± 0.168	0.431 ± 0.129	0.481 ± 0.142	0.397 ± 0.149
AUC	0.810 ± 0.099	0.800 ± 0.101	0.825 ± 0.075	0.698 ± 0.125
Kappa	0.285 ± 0.155	0.291 ± 0.110	0.351 ± 0.142	0.215 ± 0.166

Table S2. SVM cross-database performance for Wake and Sleep classification. The training database is UCDSADB database with a single random seed. For each database and each performance measurement, we report the mean ± standard deviation of all subjects.

	CGMH-training	CGMH-validation	DREAMS	UCDSADB
TP	77 ± 45	77 ± 44	110 ± 63	99 ± 55
FP	192 ± 58	167 ± 60	232 ± 83	179 ± 67
TN	421 ± 62	409 ± 92	535 ± 98	417 ± 105
FN	25 ± 33	41 ± 44	47 ± 35	58 ± 44
SE (%)	79.6 ± 15.3	71.9 ± 17.8	70.4 ± 13.3	61.4 ± 18.8
SP (%)	69.3 ± 7.0	72.0 ± 6.5	70.5 ± 7.7	70.1 ± 6.2
Acc (%)	69.7 ± 6.8	70.2 ± 5.6	69.6 ± 6.7	68.2 ± 6.5
PR (%)	29.6 ± 17.7	32.8 ± 18.6	33.1 ± 19.0	34.4 ± 17.7
F1	0.398 ± 0.168	0.409 ± 0.149	0.420 ± 0.163	0.420 ± 0.170
AUC	0.815 ± 0.107	0.783 ± 0.120	0.771 ± 0.092	0.707 ± 0.113
Kappa	0.263 ± 0.156	0.255 ± 0.136	0.266 ± 0.161	0.241 ± 0.157

SI.3.4 For the classification of Wake, REM and NREM

We consider the models trained on DREAMS and UCDSADB for the Wake, REM and NREM classification. The results are shown in Tables S7 and S8 respectively. We observe that the overall performances are consistent, and +P of NREM is higher than other classes as expected.

SI.3.5 Subsampling effect

We further evaluate the impact of the subsampling process. We repeat all the above experiments with 20 different random seeds. For each database, each performance measurement and each random seed, we record the mean of all subjects. Then we report the mean ± standard deviation of these 20 results. Specifically, we take the random seeds $1 \sim 20$ in MATLAB, which leads to 20 different training datasets after the subsampling scheme. We observe that although subsampling process was utilized in the training procedure, the performance of each SVM model is stable in the sense that all performance measurements have small standard deviation below 1%. See Tables S9 to S17.

Table S3. (Only sub-level sets filtration) SVM cross-database performance of subjects for Wake and Sleep classification with a single random seed. The training database is CGMH-training. For each database and each performance measurement, we report the mean ± standard deviation of all subjects.

	CGMH-training	CGMH-validation	DREAMS	UCDSADB
TP	79 ± 44	81 ± 49	106 ± 58	87 ± 45
FP	153 ± 50	129 ± 48	178 ± 62	154 ± 61
TN	479 ± 71	465 ± 106	611 ± 114	469 ± 118
FN	29 ± 36	44 ± 47	57 ± 47	78 ± 56
SE (%)	77.9 ± 15.0	71.8 ± 17.0	67.1 ± 16.5	53.1 ± 17.2
SP (%)	76.3 ± 6.2	79.0 ± 5.1	77.9 ± 5.5	75.3 ± 6.0
Acc (%)	75.4 ± 5.6	76.0 ± 4.7	75.0 ± 5.4	70.2 ± 6.0
PR (%)	34.4 ± 18.6	38.8 ± 19.2	37.5 ± 18.5	35.3 ± 17.9
F1	0.441 ± 0.161	0.460 ± 0.137	0.451 ± 0.149	0.399 ± 0.153
AUC	0.834 ± 0.086	0.827 ± 0.092	0.791 ± 0.095	0.691 ± 0.110
Kappa	0.323 ± 0.148	0.329 ± 0.123	0.315 ± 0.154	0.230 ± 0.147

Table S4. (Only VR-complex filtration) SVM cross-database performance of subjects for Wake and Sleep classification with a single random seed. The training database is CGMH-training. For each database and each performance measurement, we report the mean ± standard deviation of all subjects.

	CGMH-training	CGMH-validation	DREAMS	UCDSADB
TP	64 ± 35	66 ± 39	82 ± 42	69 ± 40
FP	150 ± 45	130 ± 53	200 ± 59	159 ± 54
TN	462 ± 69	445 ± 99	567 ± 103	437 ± 110
FN	39 ± 38	52 ± 44	74 ± 50	88 ± 47
SE (%)	66.9 ± 12.8	61.7 ± 15.2	55.2 ± 12.1	42.4 ± 12.4
SP (%)	75.9 ± 5.3	78.1 ± 5.8	74.3 ± 3.9	73.2 ± 5.3
Acc (%)	73.5 ± 4.6	73.7 ± 4.9	70.2 ± 3.5	66.8 ± 3.9
PR (%)	30.6 ± 16.6	34.7 ± 20.2	29.9 ± 14.9	29.5 ± 17.1
F1	0.385 ± 0.135	0.400 ± 0.138	0.362 ± 0.111	0.327 ± 0.132
AUC	0.782 ± 0.069	0.756 ± 0.107	0.697 ± 0.096	0.609 ± 0.091
Kappa	0.256 ± 0.110	0.259 ± 0.124	0.199 ± 0.093	0.136 ± 0.099

SI.3.6 Higher Dimensional Features

We further consider the impact of $\mathcal{P}_2(R_{120,1}(\mathbf{x}^{(k,j)}))$ on datasets DREAMS and UCDSADB. We integrate the feature $\mathcal{P}_2(R_{120,1}(\mathbf{x}^{(k,j)}))$ into $\mathcal{P}_0(\mathbf{x}^{(k,j)})$, $\mathcal{P}_0(R_{120,1}(\mathbf{x}^{(k,j)}))$, and $\mathcal{P}_1(R_{120,1}(\mathbf{x}^{(k,j)}))$. See Tables S18 and S19 for the results. The experiment setting follows Tables S1 and S2. Because of the computational difficulties, the testing datasets in Tables S18 and S19 are UCDSADB and DREAMS respectively.

REFERENCES

Edelsbrunner H, Harer J. Computational topology. an introduction. *Amer. Math. Soc., Providence, Rhode Island* (2010).

Munkres JR. *Elements of Algebraic Topology* (Westview Press) (1993).

Takens F. Detecting strange attractors in turbulence. Rand D, Young LS, editors, *Dynamical Systems and Turbulence* (Springer Berlin Heidelberg), *Lecture Notes in Mathematics*, vol. 898 (1981), 366–381.

Stark J, Broomhead D, Davies M, Huke J. Takens embedding theorems for forced and stochastic systems. *Nonlinear Analysis: Theory, Methods & Applications* **30** (1997) 5303–5314.

Table S5. SVM cross-database performance for REM and NREM classification. The training database is DREAMS with a single random seed. The subject #9 in CGMH-validation and the subject #24 in UCDSADB were dropped because they do not have REM epochs. For each database and each performance measurement, we report the mean ± standard deviation of all subjects.

	CGMH-training	CGMH-validation	DREAMS	UCDSADB
TP	75 ± 31	68 ± 30	102 ± 32	65 ± 35
FP	130 ± 33	121 ± 47	155 ± 53	150 ± 34
TN	382 ± 66	376 ± 90	472 ± 94	356 ± 70
FN	25 ± 24	21 ± 16	37 ± 23	48 ± 36
SE (%)	76.8 ± 15.6	77.4 ± 16.7	74.8 ± 13.1	58.3 ± 18.0
SP (%)	74.6 ± 4.9	76.4 ± 6.4	75.6 ± 5.2	70.2 ± 6.1
Acc (%)	74.6 ± 6.0	75.3 ± 8.0	75.3 ± 5.9	67.8 ± 6.9
PR (%)	36.2 ± 12.4	38.1 ± 17.9	40.6 ± 12.2	29.5 ± 15.1
F1	0.479 ± 0.132	0.483 ± 0.155	0.519 ± 0.122	0.373 ± 0.155
AUC	0.823 ± 0.109	0.834 ± 0.109	0.819 ± 0.095	0.683 ± 0.131
Kappa	0.343 ± 0.145	0.353 ± 0.166	0.375 ± 0.147	0.197 ± 0.161

Table S6. SVM cross-database performance for REM and NREM classification. The training database is UCDSADB with a single random seed. The subject #9 in CGMH-validation and the subject #24 in UCDSADB were dropped because they do not have REM epochs. For each database and each performance measurement, we report the mean ± standard deviation of all subjects.

	CGMH-training	CGMH-validation	DREAMS	UCDSADB
TP	60 ± 29	52 ± 23	75 ± 26	73 ± 40
FP	114 ± 32	108 ± 38	151 ± 45	117 ± 33
TN	398 ± 69	389 ± 93	478 ± 103	390 ± 70
FN	41 ± 25	36 ± 23	64 ± 32	41 ± 29
SE (%)	60.1 ± 17.9	60.3 ± 15.0	55.3 ± 15.5	65.3 ± 14.5
SP (%)	77.6 ± 5.0	78.1 ± 5.4	76.0 ± 4.5	76.9 ± 5.4
Acc (%)	74.6 ± 6.0	74.6 ± 9.0	72.0 ± 5.6	74.6 ± 5.9
PR (%)	33.8 ± 13.9	33.9 ± 14.5	33.6 ± 10.5	36.9 ± 16.2
F1	0.420 ± 0.143	0.412 ± 0.128	0.411 ± 0.115	0.452 ± 0.152
AUC	0.740 ± 0.135	0.742 ± 0.121	0.715 ± 0.113	0.773 ± 0.112
Kappa	0.278 ± 0.158	0.271 ± 0.146	0.244 ± 0.142	0.308 ± 0.152

Table S7. SVM cross-database performance for Wake, REM and NREM classification. The training database is DREAMS with a single random seed. For each database and each performance measurement, we report the mean ± standard deviation of all subjects.

	CGMH-training	CGMH-validation	DREAMS	UCDSADB
SE (%) (Wake)	63.3 ± 15.7	61.6 ± 17.7	64.8 ± 14.9	47.4 ± 14.7
SE (%) (REM)	54.7 ± 17.6	58.5 ± 19.0	59.4 ± 17.6	44.3 ± 17.6
SE (%) (NREM)	67.9 ± 9.3	69.3 ± 7.4	70.6 ± 8.2	61.3 ± 9.4
+P (%) (Wake)	34.7 ± 19.3	37.6 ± 16.4	44.6 ± 18.3	36.5 ± 17.5
+P (%) (REM)	37.3 ± 15.6	37.4 ± 18.0	42.4 ± 14.0	29.0 ± 16.5
+P (%) (NREM)	88.3 ± 8.7	85.5 ± 16.0	85.2 ± 7.8	75.9 ± 9.4
Acc (%)	64.5 ± 8.1	64.4 ± 8.3	66.9 ± 6.3	55.0 ± 8.5
Kappa	0.351 ± 0.116	0.346 ± 0.106	0.398 ± 0.104	0.227 ± 0.127

Table S8. SVM cross-database performance for Wake, REM and NREM classification. The training database is UCDSADB with a single random seed. For each database and each performance measurement, we report the mean $\pm$ standard deviation of all subjects.

	CGMH-training	CGMH-validation	DREAMS	UCDSADB
SE (%) (Wake)	68.8 ± 17.0	61.3 ± 20.0	59.6 ± 15.8	53.6 ± 13.9
SE (%) (REM)	26.8 ± 13.4	27.8 ± 18.9	23.9 ± 14.0	39.6 ± 17.8
SE (%) (NREM)	76.0 ± 7.5	76.7 ± 6.9	76.3 ± 6.9	72.6 ± 7.0
+P (%) (Wake)	33.9 ± 18.6	34.1 ± 15.8	37.1 ± 20.7	39.3 ± 17.1
+P (%) (REM)	36.7 ± 21.1	34.5 ± 16.6	34.7 ± 17.0	46.3 ± 22.0
+P (%) (NREM)	86.4 ± 9.1	83.1 ± 16.0	81.2 ± 8.6	77.1 ± 8.4
Acc (%)	67.1 ± 7.2	65.5 ± 9.8	64.7 ± 6.2	63.6 ± 7.3
Kappa	0.343 ± 11.2	0.313 ± 0.105	0.311 ± 0.105	0.314 ± 0.125

Table S9. SVM cross-database performance for Wake and Sleep classification. The training database is CGMH-training with 20 random seeds, $1 \sim 20$; that is, we train 20 SVM models.

	CGMH-training	CGMH-validation	DREAMS	UCDSADB
TP	6728 ± 26	2027 ± 14	2019 ± 10	2082 ± 17
FP	13200 ± 143	3360 ± 39	3456 ± 41	3679 ± 37
TN	41347 ± 143	12166 ± 39	11891 ± 41	11234 ± 37
FN	2423 ± 26	1149 ± 14	1119 ± 10	1850 ± 17
SE (%)	73.5 ± 0.3	63.8 ± 0.4	64.3 ± 0.3	53.0 ± 0.4
SP (%)	75.8 ± 0.3	78.4 ± 0.3	77.5 ± 0.3	75.3 ± 0.3
Acc (%)	75.5 ± 0.2	75.9 ± 0.2	75.3 ± 0.2	70.7 ± 0.1
PR (%)	33.8 ± 0.2	37.6 ± 0.2	36.9 ± 0.2	36.1 ± 0.1
F1	0.463 ± 0.001	0.474 ± 0.002	0.469 ± 0.002	0.430 ± 0.002
AUC	0.809 ± 0.000	0.781 ± 0.001	0.777 ± 0.001	0.693 ± 0.001
Kappa	0.331 ± 0.002	0.330 ± 0.002	0.323 ± 0.003	0.242 ± 0.002

Table S10. SVM cross-database performance for Wake and Sleep classification. The training database is DREAMS with 20 random seeds, $1 \sim 20$; that is, we train 20 SVM models.

	CGMH-training	CGMH-validation	DREAMS	UCDSADB
TP	6490 ± 49	1988 ± 16	2228 ± 13	2132 ± 23
FP	14590 ± 181	3730 ± 50	3458 ± 65	4128 ± 52
TN	39957 ± 181	11796 ± 50	11890 ± 65	10785 ± 52
FN	2660 ± 49	1189 ± 16	910 ± 13	1800 ± 23
SE (%)	70.9 ± 0.5	62.6 ± 0.5	71.0 ± 0.4	54.2 ± 0.6
SP (%)	73.3 ± 0.3	76.0 ± 0.3	77.5 ± 0.4	72.3 ± 0.4
Acc (%)	72.9 ± 0.2	76.4 ± 0.3	73.6 ± 0.3	68.5 ± 0.2
PR (%)	30.8 ± 0.2	34.8 ± 0.3	39.2 ± 0.4	34.1 ± 0.3
F1	0.429 ± 0.002	0.447 ± 0.003	0.505 ± 0.003	0.418 ± 0.003
AUC	0.781 ± 0.002	0.747 ± 0.003	0.808 ± 0.001	0.679 ± 0.002
Kappa	0.286 ± 0.002	0.293 ± 0.004	0.366 ± 0.004	0.218 ± 0.004

Table S11. SVM cross-database performance for Wake and Sleep classification. The training database is UCDSADB database with 20 random seeds, $1 \sim 20$; that is, we train 20 SVM models.

	CGMH-training	CGMH-validation	DREAMS	UCDSADB
TP	6814 ± 51	2050 ± 27	2176 ± 21	2464 ± 16
FP	16909 ± 219	4412 ± 67	4613 ± 62	4467 ± 54
TN	37638 ± 219	11114 ± 67	10734 ± 62	10445 ± 54
FN	2336 ± 51	1126 ± 27	962 ± 21	1468 ± 16
SE (%)	74.5 ± 0.6	64.6 ± 0.9	69.3 ± 0.7	62.7 ± 0.4
SP (%)	69.0 ± 0.4	71.6 ± 0.4	69.9 ± 0.4	70.1 ± 0.4
Acc (%)	69.8 ± 0.3	69.8 ± 0.3	68.7 ± 0.6	68.5 ± 0.2
PR (%)	28.7 ± 0.2	31.7 ± 0.3	32.1 ± 0.3	35.6 ± 0.2
F1	0.415 ± 0.002	0.425 ± 0.004	0.438 ± 0.003	0.454 ± 0.001
AUC	0.784 ± 0.002	0.733 ± 0.004	0.758 ± 0.003	0.716 ± 0.001
Kappa	0.261 ± 0.003	0.256 ± 0.005	0.269 ± 0.005	0.255 ± 0.002

Table S12. SVM cross-database performance for REM and NREM classification. The training database is CGMH-training with 20 random seeds, $1 \sim 20$; that is, we train 20 SVM models.

	CGMH-training	CGMH-validation	DREAMS	UCDSADB
TP	6731 ± 31	1776 ± 8	1877 ± 10	1558 ± 10
FP	10168 ± 98	2890 ± 39	2817 ± 35	3272 ± 40
TN	35446 ± 98	10338 ± 39	9749 ± 35	8921 ± 40
FN	2202 ± 31	522 ± 8	904 ± 10	1161 ± 10
SE (%)	75.4 ± 0.4	77.3 ± 0.4	67.5 ± 0.4	57.3 ± 0.4
SP (%)	77.7 ± 0.2	78.2 ± 0.3	77.6 ± 0.3	73.2 ± 0.3
Acc (%)	77.3 ± 0.1	75.8 ± 0.2	71.3 ± 0.3	70.3 ± 0.2
PR (%)	39.8 ± 0.1	38.1 ± 0.3	40.0 ± 0.2	32.3 ± 0.2
F1	0.521 ± 0.001	0.510 ± 0.003	0.502 ± 0.002	0.413 ± 0.002
AUC	0.836 ± 0.000	0.847 ± 0.001	0.796 ± 0.001	0.708 ± 0.001
Kappa	0.391 ± 0.001	0.389 ± 0.004	0.356 ± 0.003	0.234 ± 0.003

Table S13. SVM cross-database performance for REM and NREM classification. The training database is DREAMS database with 20 random seeds, $1 \sim 20$; that is, we train 20 SVM models. .

	CGMH-training	CGMH-validation	DREAMS	UCDSADB
TP	6608 ± 49	1747 ± 18	2020 ± 17	1533 ± 21
FP	11235 ± 216	3171 ± 66	3032 ± 68	3502 ± 66
TN	34379 ± 216	10057 ± 66	9534 ± 17	8692 ± 66
FN	2325 ± 49	551 ± 18	761 ± 17	1186 ± 21
SE (%)	74.0 ± 0.6	76.0 ± 0.8	72.6 ± 0.6	56.4 ± 0.8
SP (%)	75.4 ± 0.5	76.0 ± 0.5	75.9 ± 0.5	71.3 ± 0.5
Acc (%)	75.1 ± 0.3	76.0 ± 0.4	75.3 ± 0.4	68.6 ± 0.4
PR (%)	37.0 ± 0.3	35.5 ± 0.4	40.0 ± 0.4	30.5 ± 0.3
F1	0.494 ± 0.002	0.484 ± 0.005	0.516 ± 0.003	0.395 ± 0.004
AUC	0.815 ± 0.002	0.829 ± 0.006	0.816 ± 0.001	0.684 ± 0.004
Kappa	0.352 ± 0.004	0.354 ± 0.006	0.368 ± 0.004	0.208 ± 0.005

Table S14. SVM cross-database performance for REM and NREM classification. The training database is UCDSADB with 20 random seeds, $1 \sim 20$; that is, we train 20 SVM models.

	CGMH-training	CGMH-validation	DREAMS	UCDSADB
TP	5340 ± 77	1351 ± 23	1492 ± 24	1758 ± 19
FP	10351 ± 287	3044 ± 100	3368 ± 104	2869 ± 82
TN	35263 ± 287	9522 ± 100	9198 ± 104	9324 ± 82
FN	3593 ± 77	947 ± 23	1289 ± 24	961 ± 19
SE (%)	59.8 ± 0.9	58.8 ± 1.0	53.6 ± 0.9	64.7 ± 0.7
SP (%)	77.3 ± 0.6	77.3 ± 0.7	75.8 ± 0.8	76.5 ± 0.7
Acc (%)	74.4 ± 0.5	74.5 ± 0.6	71.8 ± 0.6	74.3 ± 0.4
PR (%)	34.0 ± 0.5	31.0 ± 0.7	40.8 ± 0.6	38.0 ± 0.5
F1	0.434 ± 0.004	0.406 ± 0.007	0.408 ± 0.005	0.479 ± 0.002
AUC	0.742 ± 0.005	0.733 ± 0.007	0.707 ± 0.006	0.771 ± 0.001
Kappa	0.284 ± 0.006	0.263 ± 0.009	0.236 ± 0.008	0.323 ± 0.004

Table S15. SVM cross-database performance for Wake, REM and NREM classification. The training database is CGMH-training with 20 random seeds, $1 \sim 20$; that is, we train 20 SVM models.

	CGMH-training	CGMH-validation	DREAMS	UCDSADB
SE (%) (Wake)	59.3 ± 0.2	53.4 ± 0.2	53.5 ± 0.2	37.3 ± 0.3
SE (%) (REM)	61.9 ± 0.2	65.2 ± 0.2	56.7 ± 0.4	47.7 ± 0.5
SE (%) (NREM)	71.3 ± 0.2	72.3 ± 0.3	71.5 ± 0.3	65.5 ± 0.4
+P (%) (Wake)	40.3 ± 0.1	45.3 ± 0.2	44.4 ± 0.1	38.2 ± 0.1
+P (%) (REM)	39.8 ± 0.2	37.4 ± 0.3	39.7 ± 0.2	28.0 ± 0.2
+P (%) (NREM)	89.5 ± 0.1	87.3 ± 0.1	83.7 ± 0.1	76.9 ± 0.1
Acc (%)	68.3 ± 0.1	68.2 ± 0.2	66.2 ± 0.1	57.0 ± 0.2
Kappa	0.402 ± 0.001	0.395 ± 0.002	0.372 ± 0.002	0.240 ± 0.001

Table S16. SVM cross-database performance for Wake, REM and NREM classification. The training database is DREAMS with 20 random seeds, $1 \sim 20$; that is, we train 20 SVM models.

	CGMH-training	CGMH-validation	DREAMS	UCDSADB
SE (%) (Wake)	60.1 ± 0.3	53.9 ± 0.4	62.9 ± 0.4	52.4 ± 0.4
SE (%) (REM)	53.8 ± 0.4	56.8 ± 0.5	57.9 ± 0.5	40.7 ± 0.6
SE (%) (NREM)	67.7 ± 0.3	69.6 ± 0.3	70.0 ± 0.3	72.5 ± 0.4
+P (%) (Wake)	33.9 ± 0.2	38.8 ± 0.2	44.6 ± 0.3	40.2 ± 0.2
+P (%) (REM)	37.8 ± 0.2	34.9 ± 0.3	42.8 ± 0.3	48.1 ± 0.6
+P (%) (NREM)	88.7 ± 0.1	87.3 ± 0.1	85.4 ± 0.1	77.5 ± 0.1
Acc (%)	64.6 ± 0.2	65.4 ± 0.2	67.0 ± 0.2	63.8 ± 0.2
Kappa	0.350 ± 0.002	0.354 ± 0.002	0.400 ± 0.002	0.321 ± 0.002

Table S17. SVM cross-database performance for Wake, REM and NREM classification. The training database is UCDSADB with 20 random seeds, $1 \sim 20$; that is, we train 20 SVM models.

	CGMH-training	CGMH-validation	DREAMS	UCDSADB
SE (%) (Wake)	63.7 ± 0.4	58.3 ± 0.5	47.1 ± 1.0	43.2 ± 0.9
SE (%) (REM)	26.9 ± 0.4	26.0 ± 0.5	22.7 ± 0.4	36.7 ± 0.6
SE (%) (NREM)	75.5 ± 0.3	77.0 ± 0.3	75.5 ± 0.3	70.8 ± 0.8
+P (%) (Wake)	33.0 ± 0.2	35.0 ± 0.3	36.0 ± 0.2	35.3 ± 0.3
+P (%) (REM)	38.5 ± 0.4	34.1 ± 0.5	35.6 ± 0.5	38.5 ± 0.2
+P (%) (NREM)	86.6 ± 0.1	84.7 ± 0.2	81.6 ± 0.1	75.4 ± 0.2
Acc (%)	67.0 ± 0.1	66.9 ± 0.2	64.6 ± 0.2	60.1 ± 0.3
Kappa	0.339 ± 0.002	0.322 ± 0.003	0.308 ± 0.002	0.253 ± 0.002

Table S18. SVM cross-database performance of subjects for Wake and Sleep classification with a single random seed. The feature $\mathcal{P}_2(R_{120,1}(\mathbf{x}^{(k,j)}))$ is integrated in the experiment. The training database is Dreams. For each database and each performance measurement, we report the mean $\pm$ standard deviation of all subjects.

	DREAMS	UCDSADB
TP	111 ± 56	83 ± 46
FP	172 ± 63	163 ± 63
TN	592 ± 111	421 ± 110
FN	45 ± 40	70 ± 54
SE (%)	73.6 ± 14.2	58.1 ± 16.8
SP (%)	78.0 ± 6.0	72.1 ± 7.3
Acc (%)	76.3 ± 4.9	68.2 ± 7.7
PR (%)	39.7 ± 18.2	33.3 ± 17.8
F1	0.485 ± 0.140	0.392 ± 0.155
AUC	0.826 ± 0.075	0.696 ± 0.130
Kappa	0.357 ± 0.137	0.210 ± 0.168

Table S19. SVM cross-database performance of subjects for Wake and Sleep classification with a single random seed. The feature $\mathcal{P}_2(R_{120,1}(\mathbf{x}^{(k,j)}))$ is integrated in the experiment. The training database is UCDSADB. For each database and each performance measurement, we report the mean $\pm$ standard deviation of all subjects.

	DREAMS	UCDSADB
TP	109 ± 61	97 ± 56
FP	237 ± 84	174 ± 65
TN	527 ± 97	410 ± 107
FN	47 ± 36	57 ± 43
SE (%)	70.9 ± 12.8	60.9 ± 19.1
SP (%)	69.7 ± 7.8	70.2 ± 6.7
Acc (%)	69.1 ± 6.6	68.4 ± 7.0
PR (%)	32.7 ± 18.6	34.1 ± 17.7
F1	0.418 ± 0.160	0.418 ± 0.172
AUC	0.767 ± 0.092	0.701 ± 0.125
Kappa	0.262 ± 0.155	0.238 ± 0.163
